# Interpretive JIVE: Connections with CCA and an application to brain connectivity

**DOI:** 10.3389/fnins.2022.969510

**Published:** 2022-10-14

**Authors:** Raphiel J. Murden, Zhengwu Zhang, Ying Guo, Benjamin B. Risk

**Affiliations:** ^1^Department of Biostatistics and Bioinformatics, Rollins School of Public Health, Emory University, Atlanta, GA, United States; ^2^Department of Statistics and Operations Research, University of North Carolina, Chapel Hill, NC, United States

**Keywords:** canonical correlation analysis, data integration, functional connectivity, Human Connectome Project, joint and individual variance explained, principal component analysis, structural connectivity

## Abstract

Joint and Individual Variation Explained (JIVE) is a model that decomposes multiple datasets obtained on the same subjects into shared structure, structure unique to each dataset, and noise. JIVE is an important tool for multimodal data integration in neuroimaging. The two most common algorithms are R.JIVE, an iterative approach, and AJIVE, which uses principal angle analysis. The joint structure in JIVE is defined by shared subspaces, but interpreting these subspaces can be challenging. In this paper, we reinterpret AJIVE as a canonical correlation analysis of principal component scores. This reformulation, which we call CJIVE, (1) provides an intuitive view of AJIVE; (2) uses a permutation test for the number of joint components; (3) can be used to predict subject scores for out-of-sample observations; and (4) is computationally fast. We conduct simulation studies that show CJIVE and AJIVE are accurate when the total signal ranks are correctly specified but, generally inaccurate when the total ranks are too large. CJIVE and AJIVE can still extract joint signal even when the joint signal variance is relatively small. JIVE methods are applied to integrate functional connectivity (resting-state fMRI) and structural connectivity (diffusion MRI) from the Human Connectome Project. Surprisingly, the edges with largest loadings in the joint component in functional connectivity do not coincide with the same edges in the structural connectivity, indicating more complex patterns than assumed in spatial priors. Using these loadings, we accurately predict joint subject scores in new participants. We also find joint scores are associated with fluid intelligence, highlighting the potential for JIVE to reveal important shared structure.

## 1. Introduction

Modern biomedical and scientific studies often collect multiple datasets in which the number of variables may greatly exceed the number of participants. This is common in neuroimaging studies, where multiple neuroimaging data types, referred to as modalities, as well as behavioral and demographic data, are often collected (Mueller et al., 2005; Glasser et al., 2013). The importance of such multi-dataset studies underscores the urgent need for quantitative methods capable of simultaneous analysis of these datasets.

A fundamental goal in neuroimaging is understanding the similarities between structural connectivity (SC) and functional connectivity (FC), where FC can be quantified by cross correlations between brain region time series revealed through functional magnetic resonance imaging (fMRI) and SC by measures of anatomical connections revealed using diffusion-weighted MRI (dMRI) (Honey et al., 2009). Studies have reported that brain regions with strong SC demonstrate more reliable functional connections (Honey et al., 2009; Kemmer et al., 2018), and incorporating SC information leads to more reproducible FC network estimation (Higgins et al., 2018). However, additional research is needed to elucidate the information shared between measures of connectivity and the information unique to structural or functional connectivity.

Unsupervised methods are commonly used to reduce the dimensionality of imaging datasets, which is often a key step in the joint analysis of multi-modal imaging data. Principal Components Analysis (PCA) finds components of maximum variance. It has been used to extract eigenimages from a group of individuals (Penny et al., 2011) and as a means of dimension reduction prior to employing a supervised learning method (López et al., 2011). Independent Component Analysis (ICA) is used to find components that are as independent as possible. It is commonly used to estimate resting-state networks, or regions that share a high degree of functional coupling in resting-state fMRI (Biswal et al., 2010). Non-negative matrix factorization (NNMF) constrains components to have positive entries. NNMF was used to decompose structural images from dMRI into brain regions that consistently co-varied across individuals (Sotiras et al., 2015). Auto-encoders (AEs) use neural networks for unsupervised dimension reduction. AEs have been used to learn latent feature representations from gray matter volumes extracted from structural MRI images, intensities from 18-fluoro-deoxyglucose positron emissions tomography (FDG-PET), and cerebrospinal fluid biomarkers (Suk et al., 2015). Recently, increasing attention has been paid to data integration and data fusion methods (Sui and Calhoun, 2016), which may provide insight into the relationship between structural and functional MRI without imposing a priori spatial constraints.

Statistical approaches to data integration date back to the 1930s with canonical correlation analysis (CCA) (Hotelling, 1936). Smith et al. (2015) used PCA and CCA to integrate fMRI and behavioral data from the Human Connectome Project (HCP). Recently, novel methods that assess the shared structure between datasets have arisen (Li et al., 2009; Witten et al., 2009), including several which also explore structure unique to each dataset (Lock et al., 2013; Zhou et al., 2016; Feng et al., 2018; Gaynanova and Li, 2019; Shu et al., 2020). A recent application of CCA developed a novel approach to jointly analyze functional and structural connectomes while assessing differences between groups of participants (Zhang et al., 2021).

Joint and Individual Variation Explained (JIVE) is an unsupervised method that has been used in neuroimaging (Yu et al., 2017; Zhao et al., 2019), genetic data (O'Connell and Lock, 2016; McCabe et al., 2020), and for other applications (Lock et al., 2013). JIVE is similar to PCA in that subject scores are extracted, but unlike PCA, JIVE estimates scores that are shared across datasets (joint scores) and scores that are unique to each dataset (individual scores). Common and orthogonal basis extraction (COBE), which is closely related to JIVE (Zhou et al., 2016), was applied to multi-subject resting-state correlation matrices where individual structure was used in connectome fingerprinting (Kashyap et al., 2019). Throughout the remainder of this manuscript, we will refer to the JIVE implementation in Lock et al. (2013) and the follow-up paper O'Connell and Lock (2016) as R.JIVE. An alternative algorithm and rank-estimation routine for JIVE were recently proposed in Angle-based JIVE (AJIVE) (Feng et al., 2018). AJIVE uses matrix perturbation theory (Wedin, 1972) to determine when two similar directions of variation represent noisy estimates of the same direction, and it uses a non-iterative algorithm that can decrease computational costs.

Despite the advancement in JIVE, there are limitations that may hinder its widespread application. JIVE is formulated as a subspace decomposition with shared structure captured by equivalent score subspaces, and the results can be difficult to interpret. For instance, singular value decompositions (SVDs) of joint matrices (called block-specific scores) result in subject scores that differ across datasets. The relative importance of the components of the estimated joint subspace requires an alternative representation. If JIVE is used for biomarker development, as in Sandri et al. (2018), researchers may want to estimate a subject score for a new patient, which can then be used to classify their risk. Additionally, simulation studies examining the accuracy of the rank selection procedures and estimated components are needed to provide guidance to scientific applications.

Our contributions are the following.

- We provide an intuitive view of AJIVE as averaging the canonical variables from the canonical correlation analysis of the principal component scores. We present a permutation test for the joint structure, and we call this alternative perspective and permutation test Canonical JIVE (CJIVE). The use of the phrase “interpretive JIVE” in the title of this manuscript emphasizes how we re-interpret the JIVE framework, and it is a play on the phrase “interpretive dance” and the original meaning of the jive dance from the 1930s.
- We evaluate three methods for predicting joint scores in new subjects, and demonstrate that these methods are effective at predicting joint scores in new subjects.
- Simulation studies show that, in AJIVE and CJIVE, overestimating the signal ranks can generally lead to underestimation of the joint ranks. AJIVE and CJIVE tend to outperform R.JIVE when the joint signal is small.
- We apply JIVE to the integration of functional and structural connectivity using a state-of-the-art pipeline applied to 998 subjects from the Human Connectome Project. JIVE reveals new insights into the shared variation, in particular revealing relationships that go beyond conventional spatial priors. We accurately predict joint subject scores in new subjects, and joint scores are related to fluid intelligence.

Section 2 describes the statistical methodology employed in AJIVE, R.JIVE, and sparse CCA (sCCA), and introduces CJIVE. Section 3 conducts simulation studies. Section 4 analyzes the HCP data. We discuss our findings and recommendations in Section 5.

## 2. Statistical methodology

A table of the notation we will use is given in Table 1.

**Table 1:** Notation used throughout the manuscript.

**Symbol**	**Definition or use**
*k*	Used to index the data blocks
**X**_*k*_	*kth* data block
*n*	Sample size
*p*_*k*_	Number of features (i.e., columns) in data block *k*
**J**_*k*_	Joint signal from *kth* data block
**A**_*k*_	Individual (block-specific) signal from *kth* data block
**E**_*k*_	Noise signal from *kth* data block
**I**_*d*_	An identity matrix of dimension *d*
**Z**	Joint subject scores, which form a basis for the joint signal
**B**_*k*_	Individual subject scores from data block *k*, which form a basis for that block's individual signal
**W**_*Jk*_	Variable loadings onto the joint signal subspace for the *kth* data block
**W**_*Ik*_	Variable loadings onto the individual signal subspace for the *kth* data block
*r*_*k*_	Rank of the signal contained in data block *k*
*r*_*J*_	The rank of the joint signal, i.e., the number of components comprising the joint signal
*r*_*Ik*_	The rank of the individual signal for data block *k*
**U**_*k*_	Left singular vectors of data block *k*
**D**_*k*_	Diagonal matrix containing the singular values of data block *k*
**V**_*k*_	Right singular vectors of data block *k*
**C**	Concatenation of left singular vectors from both data blocks
**U**_*C*_	Left singular vectors of concatenated singular vectors
**ω**_*kj*_	Canonical loadings, which maximize correlation of *jth* PCs
**Ω**_*k*_	Concatenation of canonical loadings
σ_*Cj*_	Joint singular value of **U**_*C*_
ρ_*j*_	Canonical correlation of the *jth* joint component
${\hat{c}}_{1i}$	Predicted canonical variables
$R_{Jk}^{2}$	Proportion of total variance in data block *k* attributable to joint signal
$R_{Ik}^{2}$	Proportion of total variance in data block *k* attributable to individual signal

### 2.1. JIVE decomposition

Consider a collection of *K* data blocks/matrices, $\left\{{\text{X}}_{k}\in {\mathbb{R}}^{n\times p_{k}}:k=1,\ldots ,K\right\}$, where *n* is the number of subjects and *p*_*k*_ the number of features or variables in the *kth* dataset. Each data block can be written as **X**_*k*_ = **J**_*k*_+**A**_*k*_+**E**_*k*_, where **J**_*k*_ represents the joint signal common to both data blocks, **A**_*k*_ represents the block-individual signal, which is unique to the *k*^*th*^ data block, and **E**_*k*_ represents full-rank isotropic noise. The JIVE model assumes that each rank-reduced signal matrix **X**_*k*_−**E**_*k*_ [with rank *r*_*k*_ < min(*n, p*_*k*_)] can be decomposed into a subspace of ℝ^*n*^ that is common across **X**_*k*_ (the joint subspace) and a subspace that is unique to the *kth* dataset and orthogonal to the joint subspace (the individual subspaces) (Feng et al., 2018). In our presentation, we expand on one of three ways to represent the joint subspace, called the “common normalized score” representation in Feng et al. (2018). We emphasize this representation because it results in a correspondence between the joint components of each dataset, whereas the other representations are arguably less interpretable. The common basis, $\text{Z}\in {\mathbb{R}}^{n\times r_{J}}$, is derived from joint analysis of all data blocks, and the other, ${\text{B}}_{k}\in {\mathbb{R}}^{n\times r_{Ik}}$ from the part that remains after joint analysis, where *r*_*Ik*_ = *r*_*k*_−*r*_*J*_. Let **I**_*d*_ be the *d*×*d* identity matrix and 0 a matrix of zeros. Furthermore, let the joint and individual signal matrices of the *k*^*th*^ data block take the form **J**_*k*_ = **ZW**_*Jk*_ and **A**_*k*_ = **B**_*k*_**W**_*Ik*_, respectively. Then the JIVE model corresponds to the matrix decomposition


(1) $$\begin{array}{l} {\;\text{X}}_{k}={\;\text{ZW}}_{Jk}+{\;\text{B}}_{k}{\text{W}}_{Ik}+{\text{E}}_{k},\\ {\text{ subject to }\text{B}}_{k}^{⊤}\text{Z}=\text{ 0},\;{\;\text{Z}}^{⊤}\text{Z}={\;\text{I}}_{r_{J}},\;{\;\text{B}}_{k}^{⊤}{\text{B}}_{k}={\;\text{I}}_{r_{Ik}}. \end{array}$$

We call **Z** joint subject scores and **W**_*Jk*_ joint variable loadings. Individual subject scores are given by **B**_*k*_ and individual variable loadings by **W**_*Ik*_. Intuitively, this decomposition is similar to a singular value decomposition on each dataset but with part of the basis constrained to be equal in the two decompositions.

In this representation, we do not enforce orthogonality between **B**_*k*_ and ${\text{B}}_{k^{\prime }}$. Later, we propose a permutation test for the joint rank, *r*_*J*_, that determines when the correlation between signal is sufficiently large to be deemed joint, but allows insignificant correlation between individual subject scores. Our proposed approach will also result in an intuitive ordering of components by the strength of evidence that they are joint. Also note that in (1), the rows of the loadings matrices **W**_*Jk*_ are not orthogonal.

For the HCP network data that we examine in Section 4, we can translate each row of the score (**Z**, **B**_*k*_) matrix into a low-dimensional vector summary of a participant's *kth* network data (e.g., FC). The joint scores **Z** summarize information that is common across modalities, while **B**_*k*_ comprise information unique to an individual modality. For instance, Section 4.3 shows that CJIVE joint scores are more strongly associated with a measure of fluid intelligence than individual scores. The *lth* row of the loading matrix **W**_*Jk*_ exhibits the magnitude with which network edges contribute to the *lth* column of the summary scores in **Z**. In Section 4.4, we examine variable loadings to develop insight into latent structures that are common within both modalities and those which are unique to each.

#### 2.1.1. R.JIVE estimation

R.JIVE uses an iterative algorithm that simultaneously estimates joint and individual matrices. Each dataset is column-centered and scaled by its Frobenius norm. In our data application, we standardize the variance of each variable prior to scaling by the Frobenius norm. The algorithm iterates between estimating the joint subspaces and individual subspaces; details are in the Web Appendix A.1.1 (Supplementary material). The ranks of the joint and individual matrices are selected using permutation tests. In the default R.JIVE implementation, the individual subspaces are orthogonal (O'Connell and Lock, 2016).

#### 2.1.2. AJIVE estimation

In AJIVE, the joint rank *r*_*J*_ is determined using principal-angle analysis (PAA) and requires user-specified signal ranks *r*_1_ = *r*_*J*_+*r*_*I*1_ and *r*_2_ = *r*_*J*_+*r*_*I*2_. The main idea is to investigate when basis vectors in the signal subspaces should be considered “noisy” estimates of the same direction. This problem can be translated into finding the singular values of the concatenated signal bases that exceed a given threshold.

For the remainder of this paper, we standardize the columns of **X**_1_ and **X**_2_ to have mean zero and variances equal to one, as commonly done in PCA. Note R.JIVE performs an additional normalization by the Frobenius norm.

First, the user specifies the ranks used in PCA of **X**_1_ and **X**_2_. Let **U**_1_ and **U**_2_ denote the *r*_1_ and *r*_2_ left singular vectors of **X**_1_ and **X**_2_. Define **C** = [**U**_1_, **U**_2_]. Let **U**_**C**_ denote the left singular vectors of **C**. Feng et al. (2018) develop two bounds to determine whether the *j*th column of **U**_**C**_ represents a joint direction of variance. These bounds are discussed in the Web Appendix A.1.2 (Supplementary material).

### 2.2. Using CCA to interpret JIVE: CJIVE

#### 2.2.1. Equivalence of estimators

We review CCA and describe how it relates to the AJIVE algorithm. For data matrices **X**_1_ and **X**_2_, CCA seeks vectors **ω**_11_ and **ω**_21_ to maximize Corr(**X**_1_**ω**_11_, **X**_2_**ω**_21_). Subsequent canonical vectors, ${\text{ω}}_{1j^{\prime }},{\text{ω}}_{2j^{\prime }}$, arise from a similar optimization problem with the additional constraint ${\text{ω}}_{1j}^{⊤}\text{Cov}\left({\text{X}}_{1}\right){\text{ω}}_{1j^{\prime }}={\text{ω}}_{2j}^{⊤}\text{Cov}\left({\text{X}}_{2}\right){\text{ω}}_{2j^{\prime }}=0$ for all *j*<*j*′. If **X**_1_ and **X**_2_ are centered and semiorthogonal matrices, then the CCA problem can be written as


(2) $$\begin{array}{l} \underset{\omega_{1j}\in {\mathbb{R}}^{p_{1}},\omega_{2j}\in {\mathbb{R}}^{p_{2}}}{\text{argmax}}\omega_{1j}^{⊤}{\text{X}}_{1}^{⊤}{\text{X}}_{2}\omega_{2j},\;j=1,\ldots ,r_{J},\;\\ \text{subject to }||\omega_{kj}||\;=\;1\text{ and }\omega_{kj}^{⊤}\omega_{kj^{\prime }}=0,\;k=1,2,\;j\neq j^{\prime }. \end{array}$$

Then the solutions to (2), which we denote as ${\hat{\text{ω}}}_{1j}$ and ${\hat{\text{ω}}}_{2j}$, are given by the left and right singular vectors of ${\text{X}}_{1}^{⊤}{\text{X}}_{2}$, which are unique up to a change in sign (Mardia et al., 1979). Additionally, $\rho_{j}=\frac{1}{n}{\hat{\text{ω}}}_{1j}^{⊤}{\text{X}}_{1}^{⊤}{\text{X}}_{2}{\hat{\text{ω}}}_{2j}$ is the *j*th canonical correlation.

Classic CCA can not be applied to *p*_*k*_>*n*. Sparse CCA is one alternative (Witten et al., 2009), and it turns out JIVE is a reduced-rank alternative. Feng et al. (2018) show that the *jth* joint subject score from AJIVE is equivalent to the average of the *jth* canonical variables of the CCA of the scores from the separate PCAs, up to scaling. Our theorem, below, formalizes their finding. A proof is provided in the Web Appendix A.3 (Supplementary material).

Theorem 2.1 *Let the columns of* **U**_1_ *and* **U**_2_ *represent orthonormal bases for the signal matrices* **X**_1_−**E**_1_ *and* **X**_2_−**E**_2_*. Let* ${\hat{z}}_{j}$ *be the* *jth* *joint subject score from AJIVE analysis. Let* ${\hat{\text{ω}}}_{1j}\in {\mathbb{R}}^{r_{1}}$ *and* ${\hat{\text{ω}}}_{2j}\in {\mathbb{R}}^{r_{2}}$ *represent the canonical vectors from the CCA of* **U**_1_, **U**_2_*. Let* σ_*Cj*_ *denote the* *jth singular value of* **C** = [**U**_1_, **U**_2_]*. Then*


$${\hat{\text{z}}}_{j}=\frac{1}{\sqrt{2}\sigma_{Cj}}({\text{U}}_{1}{\hat{\omega }}_{1j}+{\text{ U}}_{2}{\hat{\omega }}_{2j}).$$

*Additionally, the canonical correlation* $\rho_{j}=\sigma_{Cj}^{2}-1$.

**Figure 1:**
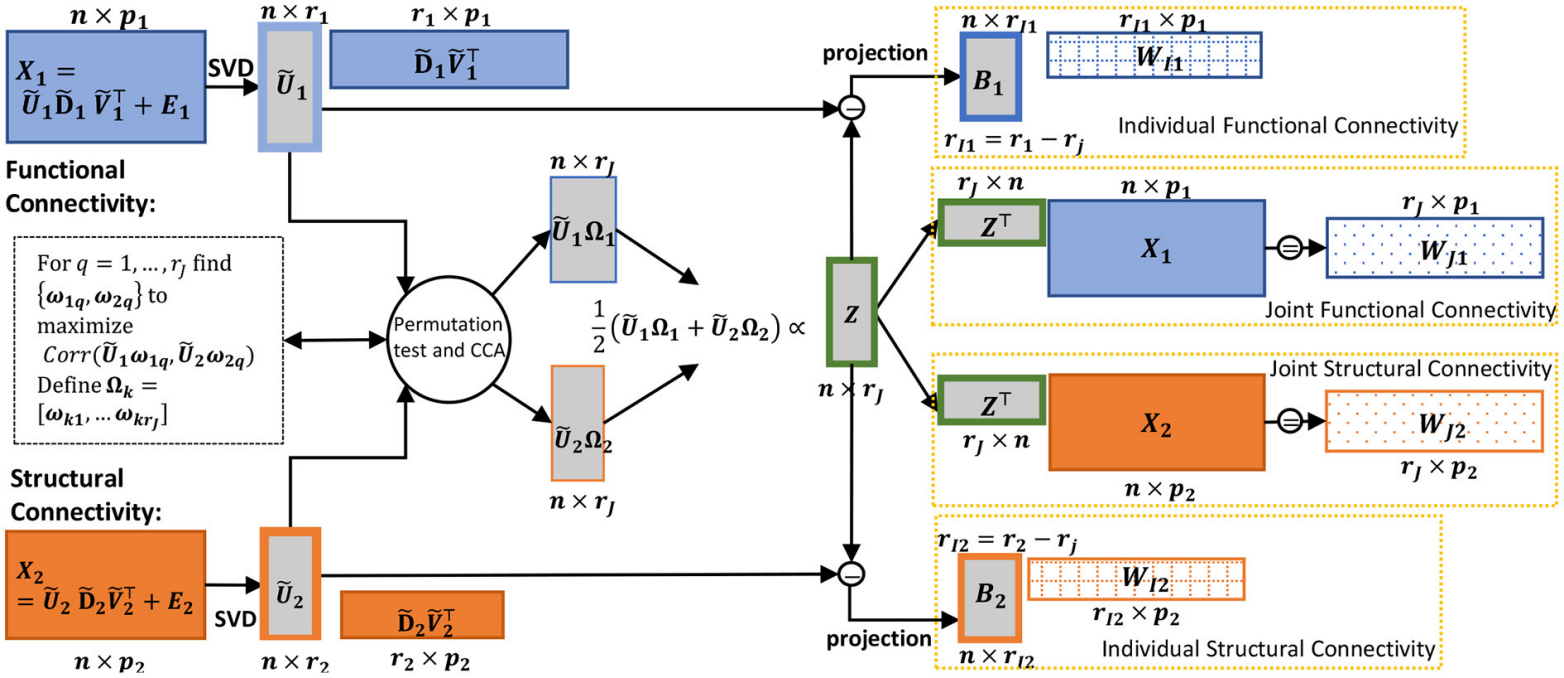
Schematic of the CJIVE decomposition for obtaining joint subject scores and loadings. Quantities specific to **X**_1_ are shown in blue; those specific to **X**_2_, orange. Gray boxes illustrate scores, with a green outline for joint scores. Checked and dotted boxes represent loadings. Steps are outlined in Algorithm 1. Separately, SVD is applied to each data block (far left) to obtain low-rank PC scores (all score matrices are shown as gray boxes). Next, CCA is applied to the PC scores with the number of components chosen using a permutation test. Joint subject scores are equivalent to a weighted average of the resultant canonical variables. Joint loadings result from the matrix product between joint subject scores and data blocks, i.e., regression of the data blocks onto joint subject scores.

In summary, the *jth* joint score vector from AJIVE is equivalent to a scaled average of the *jth* canonical variables of the principal component scores. This perspective is illustrated in Figure 1, and we define CJIVE (CCA JIVE) in the next section.

#### 2.2.2. CJIVE: Ordering, permutation test, and unique components

The CCA perspective on the signal subspaces provides a useful way to interpret the joint components. We view the canonical correlations defined in Theorem 2.1 as a measure of the strength of the corresponding joint component, which provides an ordering.

This motivates the use of a permutation test of the canonical correlations of the PCs. For *b* = 1, …, *n*_*perms*_, let ${\text{U}}_{2}^{\left(b\right)}$ represent a copy of **U**_2_ with the rows permuted so that they no longer represent the same ordering of participants as in **U**_1_. We then obtain the null distribution of the canonical correlations from the max of the singular values of ${\text{U}}_{1}^{⊤}{\text{U}}_{2}^{\left(b\right)}$, *b* = 1, …, *n*_*perms*_. For each component, we calculate a *p*-value as the proportion of maximal null correlations which exceed that component's canonical correlation. By using the max across all singular values, the family-wise error rate is controlled at the specified α-level. Once we have estimated *r*_*J*_ *via* the permutation test, we calculate joint scores using the results of Theorem 2.1 and estimate the signal matrices using the same procedure in AJIVE. Algorithm 1 describes how to conduct the CJIVE procedure.

**Algorithm 1:**
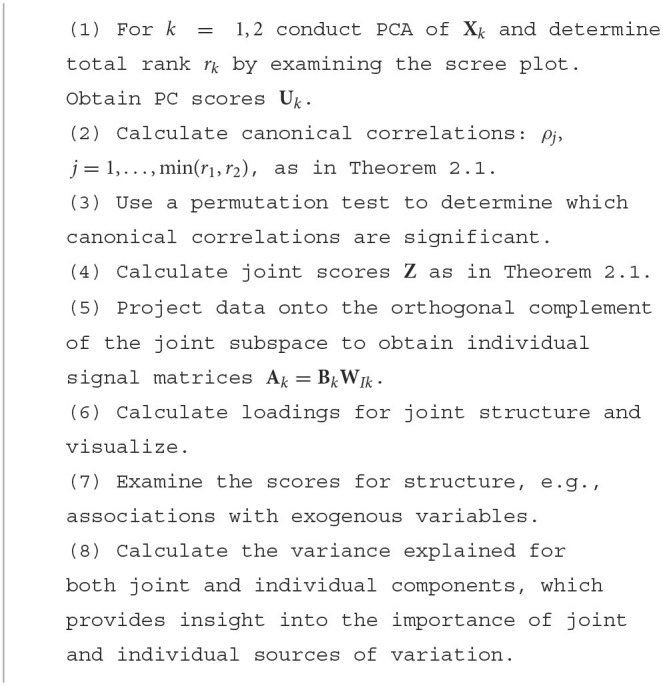
CJIVE Procedure.

Here, we summarize the CJIVE procedure depicted in Figure 1.

CJIVE provides a unique decomposition of ${\hat{\text{J}}}_{1}$ and ${\hat{\text{J}}}_{2}$ (up to sign) when the canonical correlations differ across components, as expected to occur in data. In the JIVE model given by (1), it is assumed that the joint subject score subspaces are equivalent. Then, the components are not unique. As in AJIVE (Feng et al., 2018), the joint scores represent an orthogonal basis for the joint column space. Therefore, an orthogonal transformation of these scores will result in the same joint column space.

#### 2.2.3. Predicting joint scores in new participants

An important problem is how to apply the results from JIVE analysis to a new participant. For example, if JIVE is used for biomarker development, we may want to estimate a subject score for a patient, which can then be used to classify their risk.

One straightforward way of using JIVE to predict new joint scores is to regress each new pair of observations onto the generalized inverse of joint loadings to obtain block-specific joint scores and then compute their average. Let ${\hat{\text{W}}}_{Jk}$, *k* = 1, 2, represent joint loadings from applying JIVE on the data blocks **X**_1_and**X**_2_. Let $x_{1i}\in {\mathbb{R}}^{p_{1}}$ and $x_{2i}\in {\mathbb{R}}^{p_{2}}$ be data for a new participant. Then define predicted joint scores as


$${\hat{\text{z}}}_{i}^{⊤}=({\text{x}}_{i1}^{⊤}{\hat{\text{W}}}_{J1}^{-}+{\text{ x}}_{i2}^{⊤}{\hat{\text{W}}}_{J2}^{-})/∥{\text{ x}}_{i1}^{⊤}{\hat{\text{W}}}_{J1}^{-}+{\text{ x}}_{i2}^{⊤}{\hat{\text{W}}}_{J2}^{-}∥,$$

where ${\hat{\text{W}}}_{Jk}^{-}$ represents the g-inverse of ${\hat{\text{W}}}_{Jk}$. Define this method as “G-inverse prediction.” To our knowledge, this prediction approach has not been evaluated.

R.JIVE-prediction (Kaplan and Lock, 2017) estimates subject scores in new participants using an iterative process that aims to minimize the sum of squared errors between the new data matrices and noise-decontaminated JIVE signal by alternatively estimating new subject scores with the loadings from a previous JIVE analysis.

A third approach is based on the canonical variables given in Theorem 2.1, hereafter, CJIVE-prediction. First, we predict the PC scores for a new subject; second, we estimate the canonical variables of the PC scores from each dataset; third, we sum the canonical variables and normalize to length one. Let ${\text{X}}_{k}={\text{U}}_{k}{\text{D}}_{k}{\text{V}}_{k}^{⊤}+{\text{E}}_{k},\text{for}k=1,2,$ represent a rank *r*_*k*_ SVD of **X**_*k*_. Using CCA on **U**_1_ and **U**_2_ yields matrices of canonical vectors: ${\hat{\text{Ω}}}_{1}=\left[{\hat{\text{ω}}}_{1j},\ldots ,{\hat{\text{ω}}}_{1r_{J}}\right]$ and ${\hat{\text{Ω}}}_{2}=\left[{\hat{\text{ω}}}_{2j},\ldots ,{\hat{\text{ω}}}_{2r_{J}}\right]$. The predicted estimate for each canonical variable is given by ${\hat{c}}_{1i}=x_{i1}^{⊤}{\text{V}}_{1}{\text{D}}_{1}^{-1}{\hat{\text{Ω}}}_{1}$ and ${\hat{c}}_{2i}=x_{i2}^{⊤}{\text{V}}_{2}{\text{D}}_{2}^{-1}{\hat{\text{Ω}}}_{2j}$. Then the *jth* joint score is


$${\hat{z}}_{ij}=\frac{{\hat{c}}_{1ij}+{\hat{c}}_{2ij}}{\sqrt{2(1+\rho_{j})}},$$

for *j* = 1, …, *r*_*J*_.

We apply and evaluate these three prediction methods in both the simulation study of Section 3 and analysis of the HCP data (Section 4).

## 3. Simulation study

### 3.1. Simulations comparing JIVE methods

We conduct simulation studies to address the following gaps in the current understanding of the performance of R.JIVE and AJIVE: (1) accuracy when the joint signal strength is low vs. high; (2) rank selection when the number of joint components is >1; and (3) the impact of the initial signal rank selection on joint rank selection. We use a full factorial design with the following factors:

1. The number of features in **X**_2_: with levels (a) *p*_2_ = 200 and (b) *p*_2_ = 10000,
2. Joint Variation Explained in **X**_1_: with levels (a) $R_{J1}^{2}=0.05$ and (b) $R_{J1}^{2}=0.5$,
3. Joint Variation Explained in **X**_2_: with levels (a) $R_{J2}^{2}=0.05$ and (b) $R_{J2}^{2}=0.5$.

The joint rank was 3, individual ranks were 2, and *n* = 200 in all settings. The entries of the error matrices **E**_1_ and **E**_2_ were randomly drawn from a standard Gaussian distribution. The number of features in **X**_1_ and the individual variation explained for both data blocks were held constant at *p*_1_ = 200 and $R_{I1}^{2}=R_{I2}^{2}=0.25$, respectively.

Experimental factor 1 (i.e., *p*_2_) allows us to assess the impact of *p*_*k*_ on the accuracy subspace estimation and *r*_*J*_ estimates. Factors 2 and 3 (i.e., $R_{J1}^{2}$ and $R_{J2}^{2}$) allow us to examine the impact of the joint signal's magnitude within each dataset.

For each simulation, the subject score matrix [**Z**, **B**_1_, **B**_2_] was drawn from a Bernoulli distribution, with probability 0.2 for **Z** and 0.4 for **B**_*k*_. The use of two values is similar to the toy examples from Feng et al. (2018), which used ±1. Next, we defined loading matrices **W**_*Jk*_ and **W**_*Ik*_ with entries from independent, mean 0 multivariate Gaussian distributions with covariance matrices diag(9, 4, 1) and diag(4, 1), respectively. The values along the diagonals were chosen to ensure the strength of components within each joint/individual signal diminished from first to last. Note that this set-up results in approximately orthogonal **A**_1_ and **A**_2_. In R.JIVE, we use the option enforcing this orthogonality. This set-up may favor the rank-selection procedure in AJIVE since principal angles between **A**_1_and**A**_2_ are large and corresponding singular values are unlikely to exceed the Wedin and random bounds described in Section 2.1.2.

In order to achieve the desired values of $R_{Jk}^{2}$ and $R_{Ik}^{2}$, we rescale the joint and individual matrices such that **X**_*k*_ = *d*_*k*_**J**_*K*_+*c*_*k*_**A**_*k*_+**E**_*k*_ for appropriate constants *c*_*k*_ and *d*_*k*_, as described in Web Appendix B (Supplementary material).

The chordal subspace norm is a distance metric for linear subspaces that has been generalized to matrices, say, true joint scores $\text{Z}\text{and estimated joint scores}\hat{\text{Z}}$, of possibly different ranks (Ye and Lim, 2014) and can be calculated as


$$\delta (\text{Z},\hat{\text{Z}})=\sqrt{\sum_{m=1}^{q}\sin^{2}\theta_{m}},$$

where $q=\underset{k}{\min}\left[\text{rank}\left(\text{Z}\right),\text{rank}\left(\hat{{\text{Z}}_{k}}\right)\right]$ and θ_*m*_ are the principal angles between the column space of **Z** and $\hat{\text{Z}}$. We use this metric in our simulation studies to describe the accuracy of JIVE estimates. Note when the column space of **Z** is contained in the column space of $\hat{\text{Z}}$, $\delta \left(\text{Z},\hat{\text{Z}}\right)=0$. Therefore, comparing results from different methods requires examination of rank estimates and subspace estimates.

We performed 100 simulations using the following methods: (1) AJIVE-*r*_*k*_, where we used the true total number of components *r*_*k*_ (joint rank + individual rank) as input; (2) AJIVE-Over, where the total number of components was chosen to retain 95% of the variance; (3) R.JIVE-Oracle, which uses both the true *r*_*k*_ and *r*_*J*_ as input; and (4) R.JIVE-Free, with its permutation based algorithm for choosing ranks. We also defined (5) CJIVE-*r*_*k*_ and (6) CJIVE-Over using the same approach for total signal ranks and selecting the joint rank using our permutation test with *n*_*perms*_ = 500 and α = 0.05.

To investigate the prediction methods outlined in Section 2.2.3, the subjects for each simulation were randomly divided into training and test subjects, both with sample sizes *n*/2 = 100. AJIVE, CJIVE, and R.JIVE, all with true signal ranks used as inputs, were applied on the training datasets. Subject scores were predicted for new subjects, represented by the test datasets. We then assessed performance by calculating the Pearson correlation coefficient between predicted joint scores for the test datasets and true joint scores for the same datasets for each of the *r*_*J*_ joint score components.

### 3.2. Simulation results

**Figure 2:**
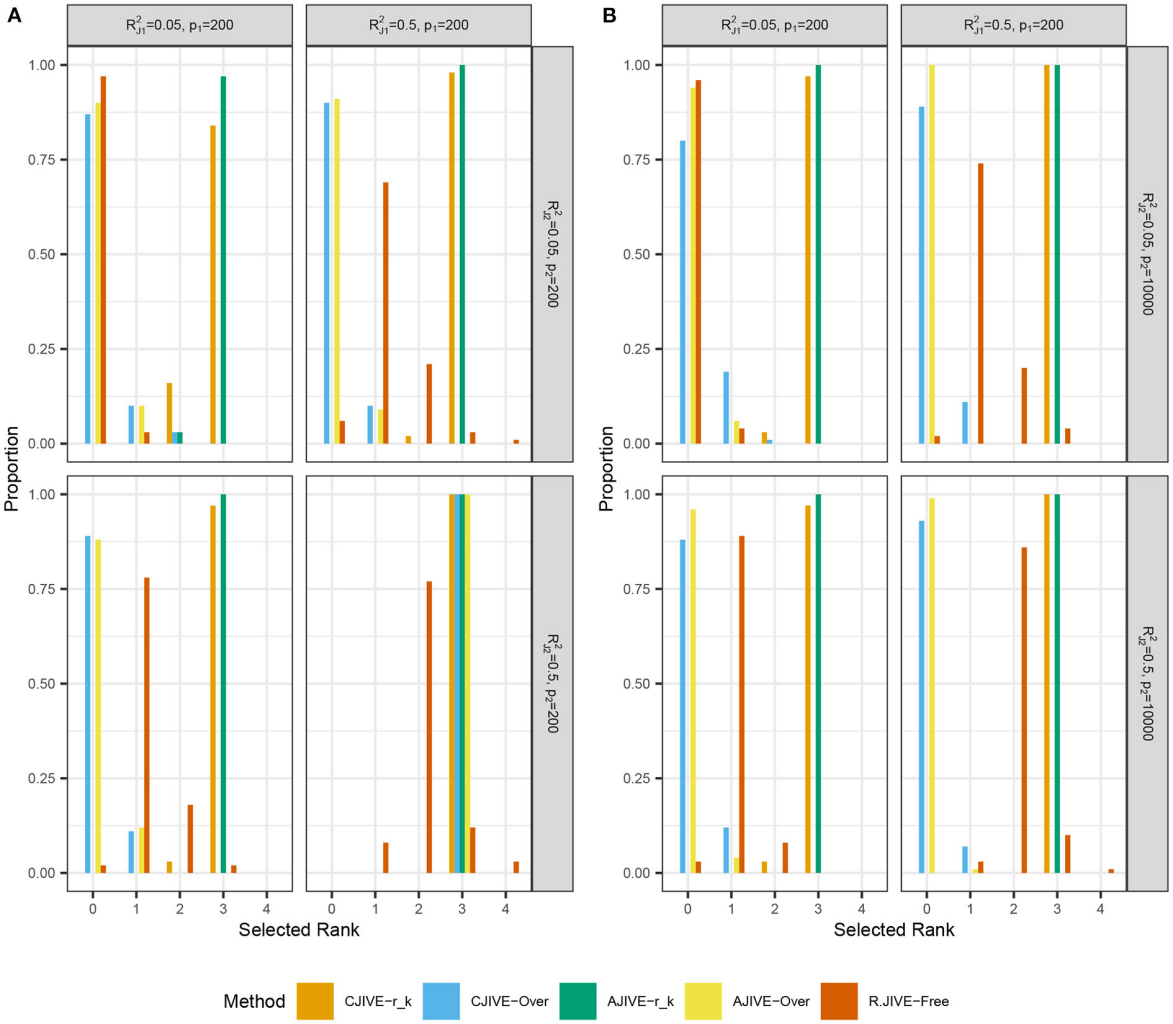
Results of simulation studies: **(A)** *p*_2_ = 200, **(B)** *p*_2_ = 10, 000. Each sub-figure shows the estimated joint signal rank for each method and combination of simulation settings. True joint rank equals 3 in all simulations. $R_{J1}^{2}$ and $R_{J2}^{2}$ represent the true joint variation controlled in simulations. We held the sample size and individual variation explained constant at *n* = 200 and $R_{I1}^{2}=R_{I2}^{2}=0.25$, respectively. Importantly, AJIVE-*r*_*k*_ and CJIVE-*r*_*k*_ are not possible in practice, as the signal ranks must all be estimated.

Figures 2A,B show that CJIVE-*r*_*k*_ and AJIVE-*r*_*k*_ chose the correct joint rank in nearly 100% of simulations in all settings except for the low-signal lower-dimensional case. Further investigation indicated the joint rank selection in AJIVE tends to be driven by the random direction bound, rather than the Wedin bound (see Web Appendix A.1.2 in Supplementary material). AJIVE-Over and CJIVE-Over both routinely underestimated the number of joint components in all scenarios except lower dimensional high-signal case. When an estimate of *r*_*k*_ is very large, the correlation between permuted datasets can be very large, such that zero joint components are significant. The joint rank estimated in R.JIVE is equal to 2 in a majority of simulations when the joint signal in both datasets is relatively large (bottom-right panels in Figures 2A,B: $R_{J1}^{2}=R_{J2}^{2}=0.5$), while it is mostly 0 or 1 in the other scenarios.

**Figure 3:**
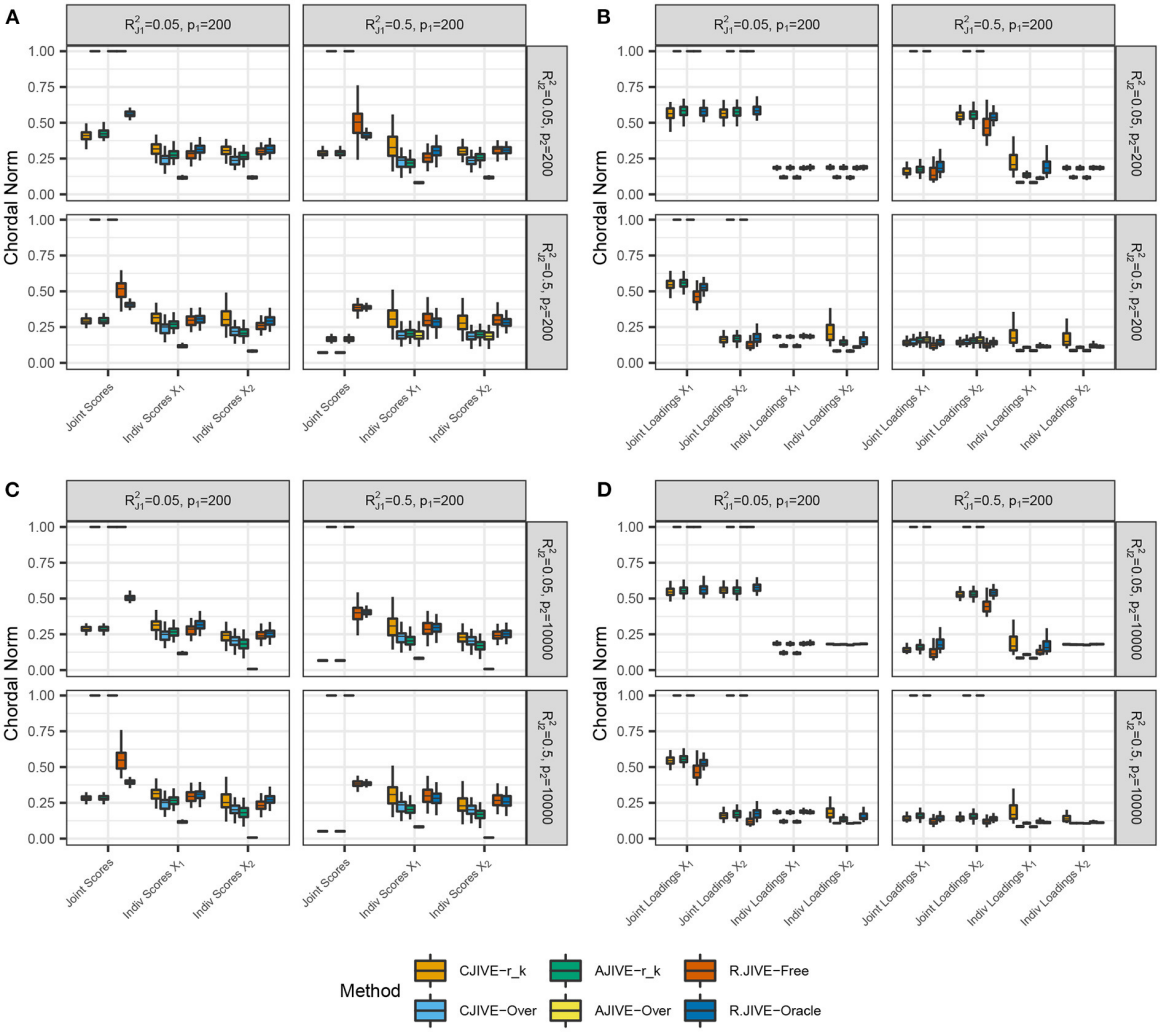
Results of simulation studies: **(A,B)** *p*_2_ = 200, **(C,D)** *p*_2_ = 10, 000. Each sub-figure exhibits boxplots of chordal norms for each of the post-JIVE measurements described in Section 2.1. Methods with chordal norms equal to 1 result when the estimated joint rank is 0 for all replicates. $R_{J1}^{2}$ and $R_{J2}^{2}$ represent the true joint variation controlled in simulations. We held the sample size, joint rank, and proportions of individual variation explained constant at *n* = 200, *r*_*J*_ = 3, and $R_{I1}^{2}=R_{I2}^{2}=0.25$, respectively. The left column **(A,C)**, show chordal norms between true and estimated subject scores. The right column **(B,D)**, show chordal norms between true and estimated variable loadings.

CJIVE-*r*_*k*_ and AJIVE-*r*_*k*_ joint score subspace errors trended less than R.JIVE, CJIVE-Over, and AJIVE-Over in all settings, as shown in Figure 3. Although the chordal distances for joint loading subspaces from R.JIVE trended less than those from AJIVE-*r*_*k*_, the lack of accurate joint rank estimates from R.JIVE may indicate that estimated subspaces partially lie within true subspaces.

To summarize, we find that CJIVE-*r*_*k*_ and AJIVE-*r*_*k*_ chose the joint rank correctly in most simulations. For both CJIVE-Over and AJIVE-Over, including too many initial signal components generally resulted in a noise-contaminated signal for each data matrix, which resulted in too few joint components or none at all. Moreover, CJIVE-*r*_*k*_ and AJIVE-*r*_*k*_ estimates of joint score and loading subspaces tended to be more accurate than both R.JIVE-Free and R.JIVE-Oracle. In simulations, AJIVE-*r*_*k*_ is equivalent to AJIVE-Scree plot because the signal rank is identified from the scree plots (Web Appendix Figure S2 in Supplementary material).

**Figure 4:**
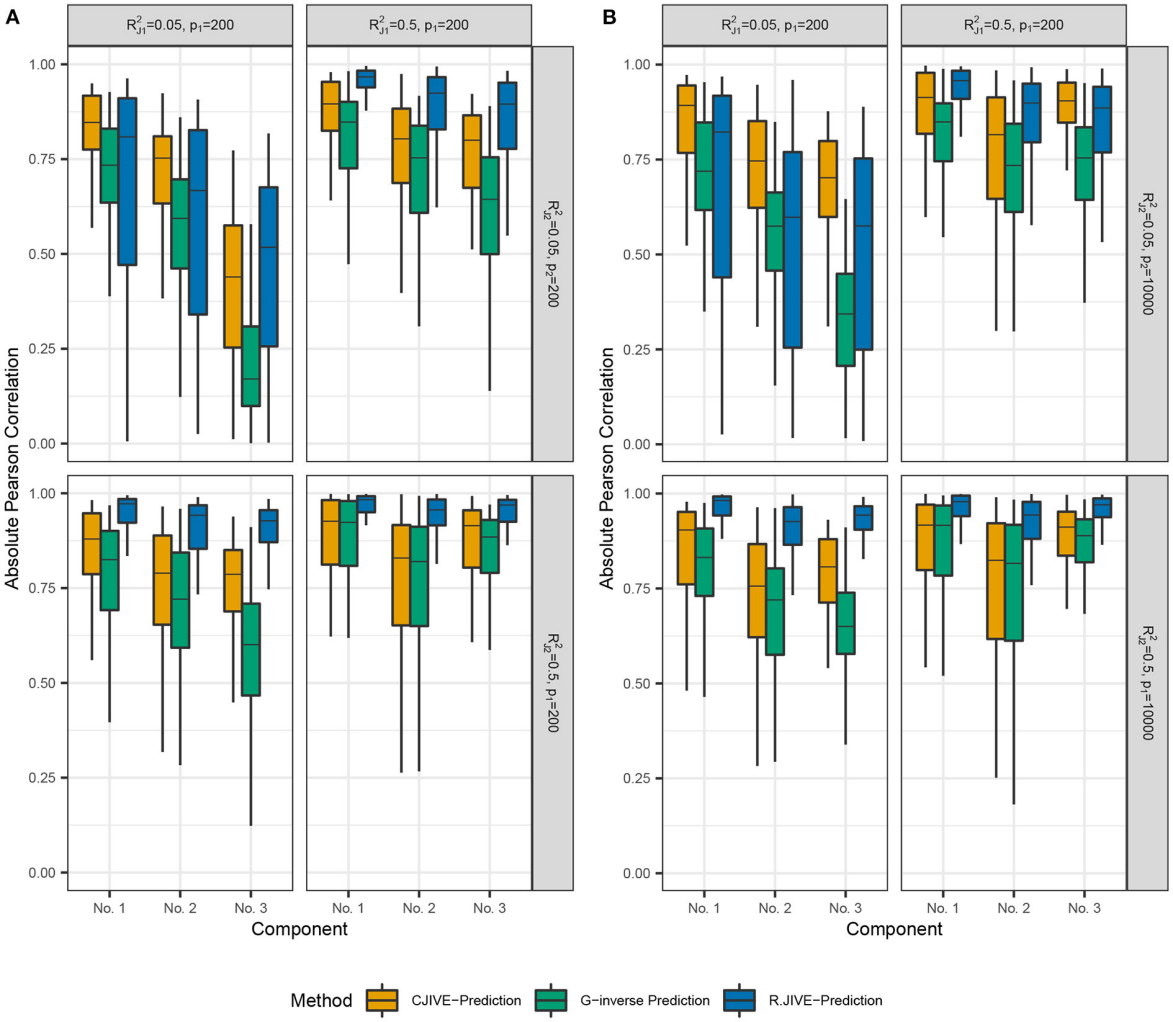
Results of simulation studies: **(A)** *p*_2_ = 200, **(B)** *p*_2_ = 10, 000. Boxplots of absolute Pearson correlations between predicted joint scores and true joint scores in simulation study. $R_{J1}^{2}$ and $R_{J2}^{2}$ represent the true joint variation controlled in simulations. We held the sample size and individual variation explained constant at *n* = 200 and $R_{I1}^{2}=R_{I2}^{2}=0.25$, respectively. **(A)** shows results for *p*_1_ = 200, *p*_2_ = 200. **(B)** shows results for *p*_1_ = 200, *p*_2_ = 10, 000. Data were simulated so that the proportion of variance attributable to the *jth* joint component in **X**_*k*_, (*k* = 1, 2;*j* = 1, …*r*_*J*_) is given by $R_{Jk}^{2}\left(\frac{r_{J}-\left(j-1\right)}{1+\cdots +r_{J}}\right)$.

Out-of-sample subject score estimates were more accurate across joint components using CJIVE-prediction compared to G-inverse prediction (Figure 4). However, R.JIVE-prediction results were most accurate when the joint signal in at least one dataset was relatively strong, i.e., $R_{Jk}^{2}=0.5$ for *k* = 1or2. The Pearson correlation coefficients tend to be close to 1, on average, for the first joint component of subject scores across all simulation settings. The third component was predicted poorly in all methods when the joint signal is relatively weak, i.e., $R_{J1}^{2}=R_{J2}^{2}=0.05.$ Recall data were simulated so that the proportion of variance attributable to the *jth* joint component in **X**_*k*_, *k* = 1, 2, *j* = 1, 2, 3 is given by $R_{Jk}^{2}=\left(\frac{3-\left(j-1\right)}{6}\right)$. Therefore, components are ordered (from highest to lowest) by the proportion of joint variation that they contribute, which may contribute to the trend in poorer prediction as *j* increased.

## 4. Joint analysis of structural and functional connectivity in the human connectome project data

### 4.1. Human connectome project data and processing

Our data application uses measures of FC and SC from *n* = 998 study participants (532 females) in the young adult Human Connectome Project (HCP). Web Appendix Table S4 in Supplementary material provides demographics. We applied R.JIVE, AJIVE, CJIVE, and sCCA to examine multivariate relationships across brain networks as measured by Fisher z-transformed correlations from rs-fMRI (FC) and log-transformed streamline counts from dMRI (SC).

HCP rs-fMRI data comprise two left-right phase encoded and two right-left phase encoded 15-min eyes-open rs-fMRI runs (Glasser et al., 2013). Each run used 2-mm isotropic voxels with 0.72 s repetition time. For each run, we calculated the average time series for each of the 68 cortical regions of interest (ROIs) from Desikan et al. (2006) plus the 19 subcortical gray-matter ROIs from Glasser et al. (2013). For each participant and pair of ROIs, the Pearson correlation was calculated, Fisher z-transformed, and then averaged across the four runs. The lower diagonal of each subject's connectivity matrix was vectorized, resulting in *p*_1_ = 3, 741.

For each HCP participant, three left-right and three right-left phase-encoded runs of dMRI from three shells of *b* = 1, 000, 2, 000, and 3, 000 s/mm^2^ with 90 directions and 6 *b*_0_ acquisitions interspersed throughout were acquired (Glasser et al., 2013). Whole-brain tractography for each participant was conducted using probabilistic tractography as detailed in Zhang et al. (2018). On average, around 10^5^ voxels occurring along the white matter/gray matter interface were identified as seeding regions for each participant. Sixteen streamlines were initiated for each seeding voxel, resulting in ~10^6^ streamlines for each participant. Nodes of the SC networks were defined from the same ROIs as the rs-fMRI. Edges were represented by the number of viable streamlines between ROIs, with viability determined by three procedures: (1) each gray matter ROI is dilated to include a small portion of white matter region; (2) streamlines connecting multiple ROIs were cut into pieces such that no streamlines pass through ROIs; and (3) apparent outliers were removed. Finally, edges where at least 99% of subjects had zero streamlines were removed, and the remaining streamline counts were log transformed. There were *p*_2_ = 3, 330 edges in the resultant SC data matrix. Plots of the mean FC and SC appear in the Web Appendix Figure S5 (Supplementary material).

### 4.2. Dimension selection and joint and individual variation explained

Both AJIVE and CJIVE with the scree-plot method for choosing total ranks estimated two joint components (Table 2), which implies that results from these methods are equivalent. Similarly, both AJIVE and CJIVE estimated 0 joint components when the total ranks were chosen to result in retaining 95% of the variation. R.JIVE with its permutation tests estimated 1 joint component.

**Table 2:** Estimated joint and total signal ranks and joint and individual variation explained in the functional connectivity (Pearson correlations) and the dMRI (streamline counts) HCP data.

**Method**		**Chosen rank**			**Variation explained**			
**Joint**	**Total**	**Joint**	**Total FC**	**Total SC**	**Joint FC**	**Individual FC**	**Joint SC**	**Individual SC**
AJIVE	Scree plot	2	7	10	0.113	0.499	0.032	0.216
	95% Var.	0	225	683	0	0.950	0	0.951
CJIVE	Scree plot	2	7	10	0.113	0.499	0.032	0.216
	95% Var.	0	225	683	0	0.950	0	0.951
R.JIVE	R.JIVE	1	54	98	0.042	0.794	0.012	0.507

FC, functional connectivity; SC, structural connectivity.

The canonical correlations were ρ_1_ = 0.31 and ρ_2_ = 0.21 using 1,000 permutations in CJIVE-Scree plot. n CJIVE-Scree plot, we also examined the breakdown of the joint variances by component: the proportion of variation attributable to joint component 1 was 0.094 in FC and 0.017 in SC (Table 2). For component 2, the values were 0.018 and 0.015, respectively.

**Figure 5:**
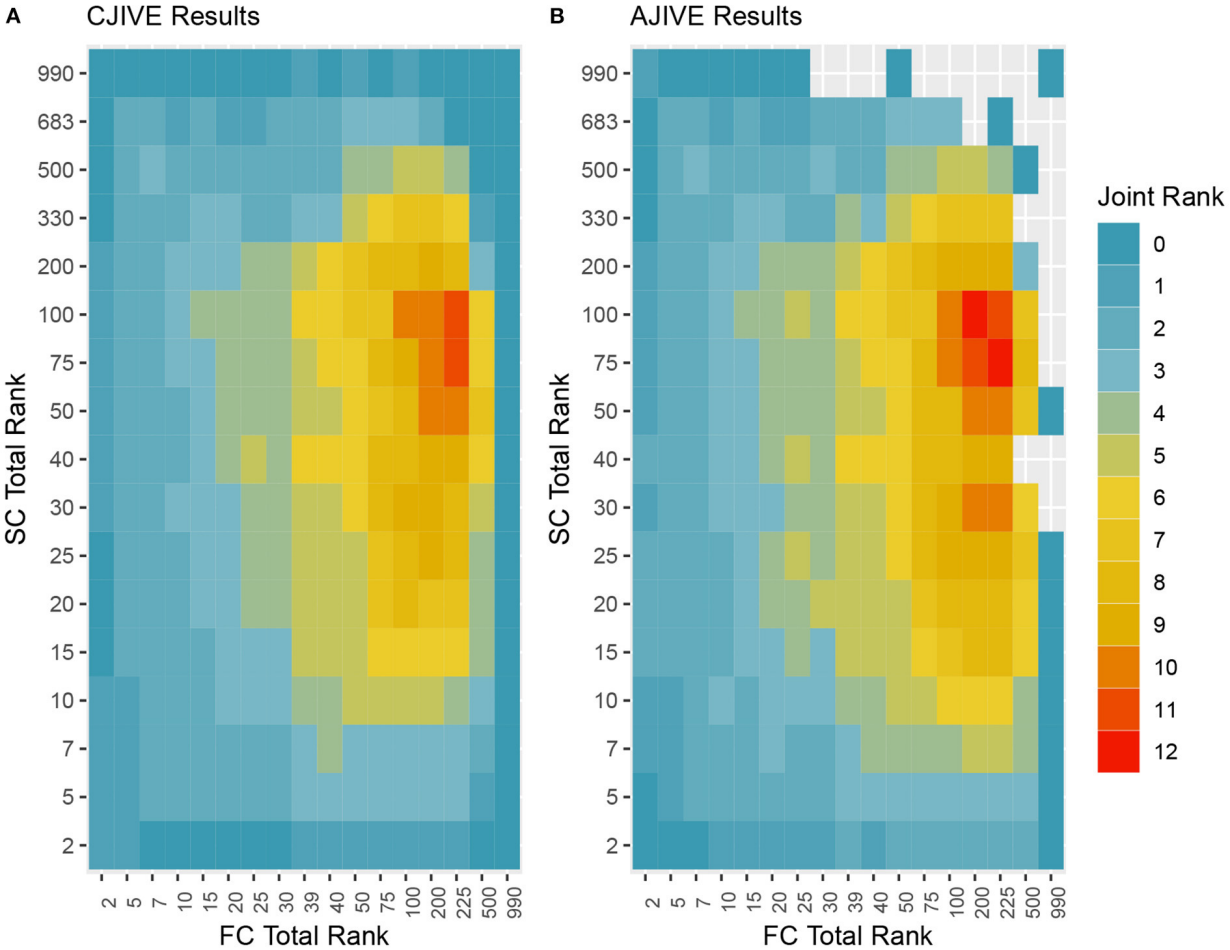
Joint ranks chosen by **(A)** CJIVE and **(B)** AJIVE. Gray boxes show when a JIVE implementation produced an error.

In addition to the previous analysis, we performed an irregular grid search to examine the impact of the signal rank selection on the estimation of the joint rank when using CJIVE and AJIVE. Specifically, we examined {2, 5, 7, 10, 15, 20, 25, 30, 39, 40, 50, 75, 100, 200, 225, 500, 990} for FC, where 39 and 225 capture 80 and 95% of the variance, respectively, and {2, 5, 7, 10, 15, 20, 25, 30, 40, 50, 75, 100, 200, 330, 500, 683, 990} for SC, where 330 and 683 capture 80 and 95% of the variance. The proportion of variation explained by the corresponding number of PCs are given in Web Appendix Table S3 (Supplementary material). The joint rank estimates for each pair of signal ranks are displayed in Figure 5. The joint rank in CJIVE and AJIVE tended to increase initially. When 80% of the variance was retained in FC and SC (*r*_*FC*_ = 39 and *r*_*SC*_ = 330), CJIVE and AJIVE estimated *r*_*J*_= 3 and 4, respectively. The joint ranks were maximized at *r*_*FC*_ = 225 and *r*_*SC*_ = 75, with CJIVE selecting *r*_*J*_ = 11 and AJIVE *r*_*J*_ = 12 (Figure 5). The joint rank estimated by AJIVE and CJIVE depends on the choice of total signal rank, but hereafter we focus on the more parsimonious representation from Figure 5, which is easier to interpret.

### 4.3. Subject scores

Joint subject scores from CJIVE-Scree plot, R.JIVE (R.JIVE using permutation tests for both joint and individual signal ranks), and sCCA, and individual scores from CJIVE-Scree plot and R.JIVE were examined for associations with fluid intelligence (gF). In the HCP, gF was measured as the number of correct responses to the Penn Progressive Matrices Test. We selected this variable as it has previously been examined in Finn et al. (2015), Smith et al. (2015), and our prior study Risk and Gaynanova (2021), and no other behavioral variables were examined. Here, AJIVE-Scree plot results are equivalent to CJIVE-Scree plot, since both methods chose two joint components. We used the R-package *rsq* to calculate the adjusted partial R-squared from the multiple regression predicting fluid intelligence from the joint and individual scores (Zhang, 2022), and then take the square root to obtain the partial correlation coefficients. We also estimated two pairs of canonical variables with sCCA. In order to compare results from sCCA to CJIVE, we averaged canonical variables across datasets to obtain a single subject score vector for each joint component. In sCCA, permutations tests resulted in sparsity parameters equal to 0.1 using the *PMA* R package (Witten et al., 2009).

Among the joint scores, CJIVE-Scree plot resulted in the highest partial correlation coefficient (*r* = 0.251). Partial correlation coefficients for individual scores (*r* = 0.248) and the overall correlation of total scores (joint + individual, *r* = 0.363) were highest in R.JIVE (Table 3). R.JIVE contained a total of 151 components while CJIVE-Scree plot included 15 components.

**Table 3:** Multiple regression of fluid intelligence onto joint subject scores estimated with CJIVE-Scree plot, R.JIVE, and sCCA. AJIVE and CJIVE are equivalent as both methods selected two joint components. Numbers in parentheses indicate the rank.

	**Partial Correlation Coefficients (ranks)**			
	**Joint**	**Indiv FC**	**Indiv SC**	**Total**
	**signal**	**signal**	**signal**	**signal**
CJIVE-Scree plot	0.251 (2)	0.091 (5)	0.080 (8)	0.278 (15)
R.JIVE	0.186 (1)	0.248 (53)	0.150 (97)	0.363 (151)
sCCA	0.200 (2)	–	–	0.200 (2)

In all three methods, only the first joint component and no individual components were significantly associated with fluid intelligence after correction for multiple comparisons (CJIVE-Scree plot: first joint component *p* = 10^−12^, Bonferroni corrected for 15 comparisons; R.JIVE: *p* = 10^−11^ for the joint component, corrected for 153 comparisons; sparse-CCA: *p* = 10^−3^ for the first joint component, corrected for 2 comparisons).

### 4.4. Variable loadings

**Figure 6:**
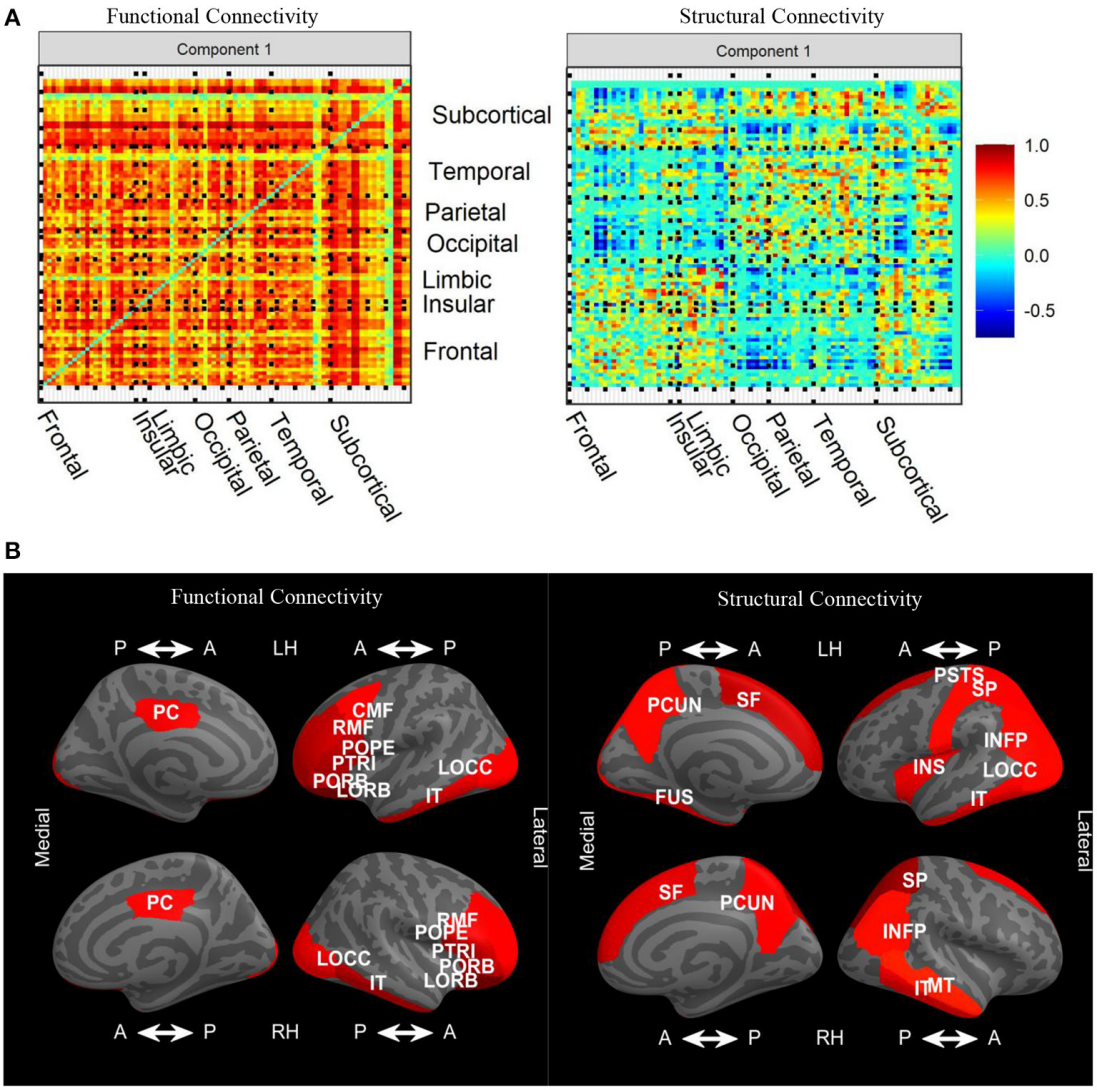
**(A)** Variable loadings for the first component of the joint signal space estimated by CJIVE and displayed on heatmaps. **(B)** Displays the top 25*th* percent of L1 norms of the variable loadings related to each cortical ROI for joint component 1. CMF, caudal middle frontal; FUS, fusiform; INFP, inferior parietal; IT, inferior temporal; INS, insula; LOCC, lateral occipital; LORB, lateral orbito-frontal; MT, middle temporal; PCUN, precuneus; PSTS, postcentral; PC, posterior cingulate; POPE, pars opercularis; PORB, pars orbitalis; PTRI, pars triangularis; RMF, rostral middle frontal; SF, superior frontal; SP, superior parietal.

Since edges from FC and SC networks comprise the features in our input data blocks, loadings are imposed onto symmetric matrices. The sign indeterminacy of the joint loadings for each component was chosen to result in positive skewness. In Figure 6A, we see that there were strong positive loadings throughout the FC. Overall, there was no clear spatial correspondence between FC and SC, and the correlation between loadings was −0.04. Instead, overall higher FC was associated with higher SC in many regions, particularly frontal-frontal and frontal-subcortical, with SC loadings in the opposite direction in certain connections between occipital, parietal, temporal, and subcortical. Plots of the joint loadings for the second component and individual loadings appear in Web Appendix Figures S3–S5 (Supplementary material).

Taking the L1 norm of each row within each loading matrix reduces the number of features to the number of nodes, which provides a more detailed examination of the patterns. In this analysis, we are particularly interested in L1 norms that are large in both the left and right hemispheres, which suggests the loadings are capturing meaningful biological structure. In the FC loadings, Figure 6B shows that the most prominent cortical regions in the first joint component correspond to ROIs from the frontal, occipital, and temporal lobes, with extensive left-right hemispheric correspondence. In the SC loadings, we again see left-right hemispheric correspondence, this time in the parietal and temporal lobes, as well as regions that did not exhibit hemispheric correspondence. L1 norms of subcortical regions (not shown) were large in the left and right accumbens, left caudate, and left putamen in both modalities. Additionally, the right putamen and right caudate were prominent in FC, while both left and right hippocampus were prominent in SC. FC and SC loadings for the individual components are depicted in Web Appendix Figures S4, S5 (Supplementary material). FC individual component 2 has large loadings on cortical to subcortical edges, and component 5 has large subcortical to subcortical loadings. SC individual component 3 has prominent loadings in both subcortical-subcortical and cortical-subcortical edges.

### 4.5. Reproducibility and prediction of new subjects

Subjects from the HCP data were split into two sets of equal sample size (*n* = 499) to examine the reproducibility of our results. We will refer to the first sub-sample as “sample A” and the second as “sample B.” CJIVE-Scree plot found *r*_*J*_ = 1 for both samples, while AJIVE-Scree plot found *r*_*J*_ = 2 for sample A and *r*_*J*_ = 1 for sample B. The correlations between the joint loadings from sample A and B were equal to 0.61 for FC and 0.65 for SC (CJIVE-Scree plot and AJIVE-Scree plot are equivalent). When a second joint component was estimated, the correlation of the FC loadings was 0.29 and the SC loadings was 0.38.

**Figure 7:**
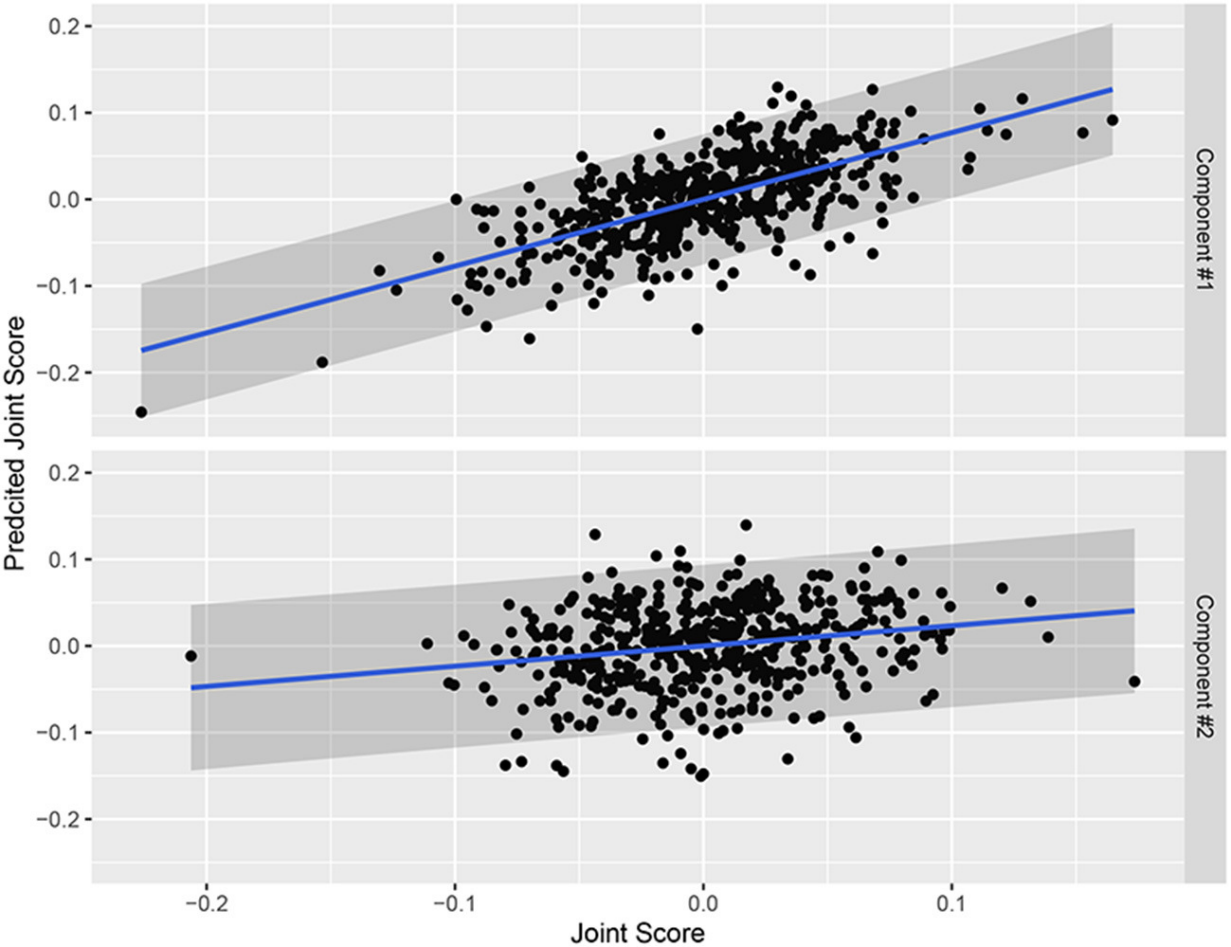
Joint subject scores using CJIVE-predict for sample B from sample A vs. joint subject scores estimated from the full CJIVE analysis of sample B. Gray bands are 95% prediction interval.

We evaluated the three prediction methods from Section 2.2.3. We compared the predicted joint scores (using the out-of-sample loadings) to those from the scores extracted from a separate analysis of sample B (Figure 7). Pearson correlations between the G-inverse predicted subject scores and CJIVE-Scree plot subject scores were 0.52 and 0.15 for components 1 and 2, respectively. Pearson correlations between subject scores estimated on sample B and those predicted for sample B using R.JIVE-predict were −0.02 and −0.03 for components 1 and 2, respectively. Using CJIVE-prediction, Pearson correlations were 0.67 and 0.22 for components 1 and 2, respectively. Similar results were achieved when CJIVE loadings from sample B data were used to predict subject scores for sample A. Recall that in simulations R.JIVE-prediction tended to outperform other methods when the joint signal was relatively large in at least one data set (Figure 4). Future research should explore the conditions that may favor R.JIVE-prediction vs. CJIVE-prediction.

### 4.6. Computation time

The computation time of CJIVE-Scree plot including the rank permutation test was 99 s. AJIVE-Scree plot with its joint rank selection took 157 s. Using pre-specified scree plot ranks and joint rank = 2, the run time for R.JIVE was over 4 h. These computation times mirrored those in our simulation study, where, on average, CJIVE was twice as fast as AJIVE and ranged from 2 to 50 times faster than R.JIVE (Web Appendix Table S1 in Supplementary material).

## 5. Discussion

We propose CJIVE, an adaptation to AJIVE which improves interpretation: (1) the joint scores are an average of the canonical variables of the principal component scores of each dataset; (2) joint scores are ordered by canonical correlations; (3) *p*-values from permutation tests indicate the significance of each joint component; (4) the proportion of variance explained for each of the joint and individual components complements this information. The joint and individual scores estimated using the CJIVE algorithm are equivalent to those estimated using AJIVE when the ranks are specified, while the R.JIVE algorithm results in different estimates of the JIVE model. CJIVE goes beyond CCA by also estimating individual components, which in some applications provides additional biological insight. Our primary contributions are improved interpretation and a faster permutation test. This provides a data-driven method to choose the joint components when conducting PCA and CCA. Simulation study results indicate that when total signal ranks are correctly specified, AJIVE and CJIVE accurately estimated the number of joint components and provided accurate estimates of the subspaces of interest.

We applied CJIVE to obtain novel insight into the relationship between structural and functional connectivity. Interestingly, we did not find a correspondence between prominent edges in FC and those in SC. However, the biological relevance of subject scores was revealed by their association with fluid intelligence, and reproducibility was demonstrated through the data splitting and prediction of the joint scores. Similarly, a recent joint analysis of FC and SC in preterm and full-term infants identified different edges in FC vs. SC (Zhang et al., 2021). Recent studies suggest that the correlation between the weighted edges in FC and SC is roughly 0.20 (Liégeois et al., 2020), which is much lower than a landmark study that contained just five subjects (Honey et al., 2009). In the current analyses, calculating the correlation between FC (averaged across subjects, as in Web Appendix Figure S3, Supplementary material) and SC was 0.22, and canonical correlations from CJIVE-Scree plot were 0.31 and 0.21. Note these approaches treat the edge as the unit of observation, and the correlations are not comparable to the variation explained in Table 2, in which the units of observation are the subject connectivity matrices and variance is across subjects. Some models assume that higher SC for a given edge leads to higher FC (Higgins et al., 2018), which we refer to as spatial priors. CJIVE allows the extraction of patterns of covariation to provide novel insight not assumed by spatial priors.

We found that CJIVE joint scores were more strongly related to fluid intelligence than joint scores from R.JIVE or sCCA. The overall correlation from R.JIVE joint and individual components was higher than CJIVE (0.36 vs. 0.28). Note R.JIVE used more components (151 vs. 15). When examining fluid intelligence and all pair-wise resting-state correlations (FC only) in the Web Explorer “HCP820-MegaTrawl,” no edges survive corrections for multiple comparisons, and using the elastic net, *r* = 0.21. Initial studies with a subsample of the HCP rs-fMRI subjects found correlations between predicted and observed fluid intelligence ranging from *r* = 0.4 to *r* = 0.5 (Finn et al., 2015; Smith et al., 2015). Previous studies did not examine the relationship between fluid intelligence, FC, and SC. Interestingly, CJIVE individual scores were not related to fluid intelligence. This may suggest that FC and SC are simultaneously associated with fluid intelligence in a manner that neither is independently. This result combined with the ability to predict out-of-sample subject scores suggests that JIVE is a possible direction for extracting biomarkers from multimodal neuroimaging. JIVE decompositions may result in fewer components than ICA or related non-Gaussian approaches. Simultaneous non-Gaussian component analysis (SING) of working memory task maps and functional connectivity matrices resulted in dozens of joint components that appeared to correspond to smaller regions with greater network specificity (Risk and Gaynanova, 2021). A possible limitation of JIVE is that the joint components may reflect brain connections involved in a variety of processes, including fluid intelligence, which may have less network specificity than ICA and non-Gaussian approaches.

In the definitions given by Chen et al. (2022), multiview analyses align datasets by subjects, whereas linked data analyses align datasets by features. Our application corresponds to multiview analysis. Kashyap et al. (2019) used Common and Orthogonal Basis Extraction (COBE), which is similar to JIVE, in a linked data application. They derived connectome fingerprints from the individual components extracted by treating each individual's FC matrix as a data block. In a single modality study from multiple groups of subjects (e.g., two sites with different subjects), one could explore common structure by applying CJIVE to the features aligned across the two sites, and then examining whether the individual components represent site-specific/batch effects. Recently, methods similar to JIVE have been proposed to conduct such analyses (Lock et al., 2022; Zhang et al., 2022). Instead of permuting subject scores, one could consider permutation tests in the feature signal subspace for testing joint components. Related, group ICA can be viewed as a linked data analysis version of AJIVE treating the space-by-time matrix from each subject as a block and including an additional step that rotates group components. Group ICA first conducts PCA on each subject's space-by-time matrix, concatenates the spatial eigenvectors, then conducts a second PCA to arrive at group components. This procedure is also used in the AJIVE algorithm, except that in group ICA the PC steps are performed on aligned features rather than aligned subjects. Group ICA then performs an additional step in which the group components are rotated to maximize their “independence,” which improves interpretability.

In practice, choosing the total signal rank remains a challenge. In simulations, the total signal rank chosen for a data block *via* R.JIVE permutation tests varied with the level of joint signal and the number of features within that block (Web Appendix Figure S1 in Supplementary material), and the estimated number of components was relatively large in the real data. Additionally, scree plots of simulated data provide a much clearer distinction between eigenvalues that correspond to signal and those lying outside the signal subspace when compared to scree plots of real data. Most pertinent to our analyses is the result that both the CJIVE and AJIVE methods for estimating the joint rank are sensitive to estimates of the total signal ranks. If *r*_*k*_ approaches *n*, the maximum correlation between permuted datasets is very high, which can lead to the estimation of zero joint components. In fact, when *r*_*k*_ = *n*, the correlation between permuted datasets equals one, and hence zero components are selected by CJIVE. The same issue occurs in AJIVE.

Further research is needed to explore connections between CJIVE and AJIVE estimates for more than two datasets. Multiset CCA (mCCA) (Li et al., 2009) extends CCA to multiple datasets by maximizing the sum of pairwise correlations. A CJIVE variant on mCCA may provide novel insights into individual structure. A related issue is that for more than two datasets, joint signal may be shared by a subset of datasets (Gaynanova and Li, 2019). When combining more than two datasets, future research should examine optimal ways of combining the canonical variables of the PC scores.

## Data availability statement

Data was provided, in part by the Human Connectome Project, WU-Minn Consortium (Principal Investigators: David Van Essen and Kamil Ugurbil;1U54MH091657) funded by the 16 NIH Institutes and Centers that support the NIH Blueprint for Neuroscience Research; and by the McDonnell Center for Systems Neuroscience at Washington University. The datasets analyzed for this study can be found at: https://www.humanconnectome.org/study/hcp-young-adult. Requests to access these datasets should be directed to Human Connectome Project, https://www.humanconnectome.org/study/hcp-young-adult.

## Author contributions

RM, ZZ, YG, and BR contributed to conception and design of the study. RM, ZZ, and BR processed the data. RM conducted the data analysis and simulation studies. RM and BR wrote the manuscript. All authors contributed to the article and approved the submitted version.

## Funding

This work was supported by R21 AG066970 to ZZ and BR, R01 MH105561 to YG, and R01 MH129855 to BR.

## Conflict of interest

The authors declare that the research was conducted in the absence of any commercial or financial relationships that could be construed as a potential conflict of interest.

## Publisher's note

All claims expressed in this article are solely those of the authors and do not necessarily represent those of their affiliated organizations, or those of the publisher, the editors and the reviewers. Any product that may be evaluated in this article, or claim that may be made by its manufacturer, is not guaranteed or endorsed by the publisher.

## Author disclaimer

The content is solely the responsibility of the authors and does not necessarily represent the official views of the National Institutes of Health.
