# Supplementary material for: Interpretive JIVE: Connections with CCA and an application to brain connectivity
Source: Front Neurosci. 2022 Oct 14;16:969510. doi: 10.3389/fnins.2022.969510 (PMC9614436; doi:10.3389/fnins.2022.969510)
Supplement: Supplementary file 1 [file Data_Sheet_1.PDF]

This appendix provides additional details and supplementary information about interpreting AJIVE analysis by using canonical correlation analysis on the estimated signal matrices (i.e. CJIVE). In Section A, we describe the AJIVE and R.JIVE algorithms for model estimation and the equivalence between AJIVE joint scores and CJIVE joint scores. In Section B, additional information about and results from the simulation study described in the main article are provided. Section C presents additional information related to the JIVE analysis of HCP data in the main article.

WEB APPENDIX A: STATISTICAL METHODOLOGY

Here we outline the algorithms of R.JIVE and AJIVE. R.JIVE employs permutation tests to estimate the joint and individual signal ranks within each of the datasets analyzed. Principal-angle analysis (PAA) is used in the AJIVE method to determine joint rank. Scree plots are recommended for choosing total rank in AJIVE, although other options are explored in the main article.

Web Appendix A.1: JIVE Methods

For a collection of K data matrices JIVE decomposes each matrix $\mathbf{X}_k$, $k = 1, \ldots, K$ into a joint signal, $\mathbf{J}_k$, individual signal, $\mathbf{A}_k$, and additive noise $\mathbf{E}_k$. Let $\mathbf{X}_k \in \mathbb{R}^{n \times p_k}$, where n is the number of subjects and p_k the number of features or variables. Let $C(\mathbf{G})$ define the column space of a matrix $\mathbf{G}$, i.e., $C(\mathbf{G}) = \{\boldsymbol{v} \in \mathbb{R}^n : \exists \mathbf{t} \ \in \mathbb{R}^p$ such that $\boldsymbol{v} = \mathbf{Gt}\}$.

$$\begin{aligned} \mathbf{X}_k = \mathbf{J}_k + \mathbf{A}_k + \mathbf{E}_k, \quad \text{where} \\ C(\mathbf{J}_k) = C(\mathbf{J}_{k'}), \text{ for all } k, k' \subset 1, \ldots, K, \\ C(\mathbf{J}_k) \perp C(\mathbf{A}_k) \text{ for } k = 1, \ldots, K, \\ \mathbb{E}(\mathbf{E}_k) = \mathbf{0}_{n \times p_k}. \end{aligned} \tag{S1}$$

Let r_k denote the signal rank of the kth dataset, r_J denote the joint rank, and r_{Ik} denote the rank of the individual subspace. Because we assume that the joint and individual signals are orthogonal, $r_k = r_J + r_{Ik}$. In this study, we focus on $K = 2$.

Web Appendix A.1.1: R.JIVE Estimation

In addition to the assumptions in (S1), R.JIVE assumes the error matrices $\mathbf{E}_k$ have independent entries. In Lock et al. (2013), two automated methods were proposed for choosing the joint rank. One of these is a permutation test; the other, a strategy that utilizes Bayesian Information Criterion (BIC). In the case that either of the automated rank choice methods is used, R.JIVE will also compute the mean squared error (MSE) between consecutive estimates of the total signal matrices $\mathbf{G}_k = \mathbf{J}_k + \mathbf{A}_k$ in an iterative process which simultaneously chooses ranks and estimates signals.

Algorithm:

1. Calculate the centered/scaled matrices $\mathbf{X}_{1,cs}$, $\mathbf{X}_{2,cs}$:

$$\mathbf{X}_{k,cs} = ||\mathbf{X}_k||_F^{-1}(\mathbf{I}_n - n^{-1}\mathbf{1}_n\mathbf{1}_n^\top)\mathbf{X}_k, \quad k = 1, 2$$

where $|| \cdot ||_F$ denotes the Frobenius norm and $\mathbf{1}_n$ is an $n \times 1$ vector of ones.

2. Estimate ranks r_J, r_1 and r_2 with n_{perm} permutations and significance level $\alpha \in (0, 1)$ (e.g., $n_{perm} = 100$ and $\alpha = 0.05$):

a. Estimate r_J:
   (1) Let λ_j be the jth singular value of $\mathbf{X} = [\mathbf{X}_{1,cs}, \mathbf{X}_{2,cs}]$ for $j = 1, \ldots, \min(n, p_1 + p_2)$
   (2) Permute the rows within each $\mathbf{X}_{k,cs}$ and calculate the singular values of the resultant concatenated matrix. Repeat n_{perm} times.
   (3) Let λ_j^{perm} be the $100(1-\alpha)$ percentile calculated from the n_{perm} samples of the jth singular value from permuted data.
   (4) Choose r_J to be the largest integer such that $\forall j \le r_J, \lambda_j > \lambda_j^{perm}$.

b. Estimate r_1 and r_2:
   (1) Let λ_{j_k} be the jth singular value of $\mathbf{X}_k$ for $k = 1, 2$ and $j_k = 1, \ldots, \min(n, p_k)$
   (2) Permute the rows separately within each column of $\mathbf{X}_k$ and calculate the singular values of the resultant matrix. Repeat n_{perm} times.
   (3) Let $\lambda_{j_k}^{perm}$ be the $100(1-\alpha)$ percentile among the j_k^{th} singular values after permutation.
   (4) Choose r_k to be the largest integer such that $\forall j_k \le r_k, \lambda_{j_k} > \lambda_{j_k}^{perm}$

3. Use the estimates of $r_J, r_1,$ and r_2 found with the permutation tests above to estimate the signal matrices $\mathbf{J} = [\mathbf{J}_1, \mathbf{J}_2]$ and $\mathbf{G} = [\mathbf{A}_1, \mathbf{A}_2]$. Loop until $\widehat{MSE} = \sum_{k=1}^{n} \sum_{l=1}^{p_1+p_2} \frac{(\hat{\mathbf{G}}^{new}[k,l] - \hat{\mathbf{G}}^{old}[k,l])^2}{n(p_1+p_2)}$ is less than a given threshold:

   a. Initialize $\mathbf{X}_J = [\mathbf{X}_{1,cs}, \mathbf{X}_{2,cs}]$ and estimate $\hat{\mathbf{J}}$ as a rank r_J SVD of $\mathbf{X}_J$.

$$\hat{\mathbf{J}} = \mathbf{U}_{r_J} \mathbf{D}_{r_J} \mathbf{V}_{r_J}^\top = [\hat{\mathbf{J}}_1, \hat{\mathbf{J}}_2],$$

   b. For $k = 1, 2$, set $\hat{\mathbf{X}}_k = \mathbf{X}_{k,cs} - \hat{\mathbf{J}}_k$ and estimate $\hat{\mathbf{A}}_k$ with a rank r_k SVD of $(\mathbf{I}_n - \mathbf{U}_{r_J}\mathbf{U}_{r_J}^\top)\hat{\mathbf{X}}_k$

$$\hat{\mathbf{A}}_k = \mathbf{U}_{r_{Ik}} \mathbf{D}_{r_{Ik}} \mathbf{V}_{r_{Ik}}^\top.$$

   c. Set $\hat{\mathbf{X}}_J = [\mathbf{X}_{1,cs}, \mathbf{X}_{2,cs}] - [\hat{\mathbf{A}}_1, \hat{\mathbf{A}}_2]$
   d. Use procedure (i) in step (b) above to re-estimate r_J using the concatenated matrix $\hat{\mathbf{X}}_J$ from step iii. Similarly, re-estimate r_1 and r_2 by procedure (ii) in step (b) above using the the matrices $\mathbf{X}_{k,cs} - \hat{\mathbf{J}}_k, k = 1, 2$.
   e. Repeat (c) loop with $\mathbf{X}_J = \hat{\mathbf{X}}_J$

Web Appendix A.1.2: AJIVE Estimation

AJIVE imposes additional constraints on the model given in equation (S1). The error matrices $\mathbf{E}_k$ both follow an isotropic error model, which implies that the energy of projection is invariant to the direction in both row and column spaces. The standard multivariate Gaussian distribution and the multivariate student t-distribution with shape matrix equal to the identity matrix are both examples of isotropic models (Feng et al. (2018)). Furthermore, individual signals' vector subspaces have null intersection: $\mathbf{A}_1 \cap \mathbf{A}_2' = \mathbf{0}$.

The number of joint components r_J (i.e. the joint rank) is determined using principal-angle analysis (PAA). Define $\mathbf{U}_k$ as the orthonormal left singular vectors of matrices $\mathbf{G}_k$, $k = 1, 2$. Then we can write an SVD of their inner product as $\mathbf{U}_1^\top \mathbf{U}_2 = \mathbf{U} \cos(\mathbf{\Theta}) \mathbf{V}^\top$, where $\mathbf{\Theta} = (\theta_1, \ldots, \theta_q)$ is the vector of principal angles between $C(\mathbf{U}_1)$ and $C(\mathbf{U}_2)$, and $\mathbf{U}, \mathbf{V}$ are the left- and right- singular vectors, respectively of the inner product $\mathbf{U}_1^\top \mathbf{U}_2$. The following theorem and subsequent lemma are used to develop a bound on the

angle between two common subspaces perturbed by isotropic error, which is complimented by a random direction bound.

THEOREM S0.1 (Wedin (1972)). *For $k = 1 \dots K$, let θ_k be the largest principal angle between the subspace spanned by $\mathbf{G}$ and that spanned by $\widetilde{\mathbf{G}}_k = \mathbf{G}_k + \mathbf{E}_k$ and denote the SVD of $\widetilde{\mathbf{G}}_k = \widetilde{\mathbf{U}}_k \widetilde{\mathbf{D}}_k \widetilde{\mathbf{V}}_k^\top$. Then*

$$\sin(\theta_k) \leq \frac{\max(||\mathbf{E}_k \widetilde{\mathbf{V}}_k||, ||\mathbf{E}_k^\top \widetilde{\mathbf{U}}_k||)}{\sigma_{\min}(\widetilde{\mathbf{D}}_k)},$$

where $\sigma_{\min}(\widetilde{\mathbf{D}}_k)$ is the minimal non-zero singular value of $\widetilde{\mathbf{G}}_k$

LEMMA S0.1 (Feng et al. (2018)). *Let ϕ be the largest principal angle between two subspaces that are each a perturbation of the common column space within $C(\widetilde{\mathbf{G}}_1)$ and $C(\widetilde{\mathbf{G}}_2)$. Suppose θ_1, θ_2 are the respective angles for $\widetilde{\mathbf{G}}_1, \widetilde{\mathbf{G}}_2$ from Theorem S0.1. Then ϕ is bounded by*

$$\sin(\phi) \leq \sin(\theta_1 + \theta_2).$$

Below we outline the algorithm employed by the AJIVE method.

1. Data blocks are centered,
$$\mathbf{X}_{k,c} = (\mathbf{I}_n - n^{-1}\mathbf{1}_n\mathbf{1}_n^\top)\mathbf{X}_k, \quad k = 1, 2$$
2. Let $r_k = r_J + r_{Ik}$ represent the total signal rank of data matrix $\mathbf{X}_{k,sc}$, $(k = 1, 2)$. Take the Singular Value Decomposition (SVD) of each data block and obtain $\mathbf{X}_k = \mathbf{U}_k \mathbf{D}_k \mathbf{V}_k^\top$. Concatenate the first r_k left-singular vectors of both datasets to form $\mathbf{J} = [\widetilde{\mathbf{U}}_1, \widetilde{\mathbf{U}}_2] \in \mathbb{R}^{n \times (r_1 + r_2)}$.
3. Principal Angle Analysis:
   a. Denote the residual left singular vectors from each SVD in step 2 above as $\mathbf{U}_k^\perp \in \mathbb{R}^{n \times (\min(p_k, n) - r_k)}$. Similarly, let $\mathbf{V}_k^\perp \in \mathbb{R}^{p_k \times (\min(p_k, n) - r_k)}$. be the residual right singular vectors from the SVDs in step 1 above.
   b. To estimate the unobserved values $||\mathbf{E}_k \widetilde{\mathbf{V}}_k||$ and $||\mathbf{E}_k^\top \widetilde{\mathbf{U}}_k||$, sample from directions orthogonal to the signal space and project the data block onto those sampled directions. For each k, sample r_k non-zero columns without replacement from $\widetilde{\mathbf{U}}_k$, denoted $\mathbf{U}_k^*$ and compute $||\mathbf{X}_k^\top \mathbf{U}_k^*||$. Perform this sampling and computation 1000 times to approximate the distribution of $||\mathbf{E}_k^\top \widetilde{\mathbf{U}}_k||$. Similarly, approximate the distribution of $||\mathbf{E}_k \widetilde{\mathbf{V}}_k||$ with a random sampling of $||\mathbf{X}_k \mathbf{V}_k^*||$ values.
   c. (*Wedin bound*) Obtain a full-rank, $\min(n, r_1 + r_2)$, SVD of $\mathbf{J} \approx \mathbf{U}_J \mathbf{D}_J \mathbf{V}_J^\top$. Let $d_{J,i}$ represent the ith singular value of $\mathbf{J}$; i.e. the ith of entry of $diag(\mathbf{D}_J)$. Then the Wedin bound is estimated by the distribution of
   $$2 - \sum_{k=1}^{2} \left(\frac{\max(||\mathbf{X}_k \mathbf{U}_k^*||, ||\mathbf{X}_k^\top \mathbf{V}_k^*||)}{\sigma_{\min}(\bar{\mathbf{D}}_k)} \right)^2,$$
   where sampled values of $||\mathbf{X}_k^\top \mathbf{U}_k^*||$ and $||\mathbf{X}_k \mathbf{V}_k^*||$ are used to approximate the unobserved values $||\mathbf{E}_k \widetilde{\mathbf{U}}_k||$ and $||\mathbf{E}_k^\top \widetilde{\mathbf{V}}_k||$, respectively, as in the procedure explained in step (b) above. The 95th percentile is used as the bound.
   d. (*Random direction bound*) The random direction bound aims to assess whether angles between directions in the proposed signal space correspond to random directions driven by noise. The distribution of principal angles generated by random subspaces is simulated as follows. Each $\widetilde{\mathbf{U}}_k$

is right-multiplied by an independent orthonormal matrix to obtain $\mathbf{U}_k^{**}$. The 95th percentile of replicates of the principal angle derived from the maximum eigenvalue of $[\mathbf{U}_1^{**}, \mathbf{U}_2^{**}]$ gives the second bound. The joint rank r_J is then chosen as the number of eigenvalues $d_{J,i}$ exceeding both bounds.

4. The first r_J left singular vectors of $\mathbf{J}$ (corresponding to the r_J singular values obtained in PAA) represent a basis of the data matrices' estimated joint column space. Use them to form an orthogonal projection operator, $\mathbf{M}_J = \mathbf{U}_J\mathbf{U}_J^\top$, and project each dataset onto the estimated joint column space spanned by $\mathbf{U}_J$ to obtain estimates of the joint signal matrices.

$$\hat{\mathbf{J}}_k = \mathbf{M}_J\mathbf{X}_{k,c}.$$

5. Lastly, the column space for each individual signal is found by computing an orthogonal projection operator onto the orthogonal complement of the joint column space with respect to the data's column space. The estimated individual signals are then given by:

$$\hat{\mathbf{A}}_k = (\mathbf{U}_k\mathbf{U}_k^\top - \mathbf{M}_J)\mathbf{X}_{k,c}.$$

Web Appendix A.2: Post AJIVE/R.JIVE Representations

As discussed in Feng et al. (2018), joint scores and loadings can be computed to represent data-specific information or common information across datasets.

Let $\hat{\mathbf{J}}_{k(R)}, \hat{\mathbf{A}}_{k(R)}$ and $\hat{\mathbf{J}}_{k(A)}, \hat{\mathbf{I}}_{k(A)}$, for $k = 1, 2$ represent the signal matrices estimated using the R.JIVE and AJIVE methods, respectively. Take their SVDs:

$$\hat{\mathbf{J}}_{k(\cdot)} = \mathbf{U}_{\hat{\mathbf{J}}_{k(\cdot)}}\mathbf{\Sigma}_{\hat{\mathbf{J}}_{k(\cdot)}}\mathbf{V}_{\hat{\mathbf{J}}_{k(\cdot)}}^\top, \quad \hat{\mathbf{I}}_{k(\cdot)} = \mathbf{U}_{\hat{\mathbf{I}}_{k(\cdot)}}\mathbf{\Sigma}_{\hat{\mathbf{I}}_{k(\cdot)}}\mathbf{V}_{\hat{\mathbf{I}}_{k(\cdot)}}^\top.$$

Estimates of data-specific joint and individual subject scores are defined as $\mathbf{U}_{\hat{\mathbf{J}}_{k(\cdot)}}\mathbf{\Sigma}_{\hat{\mathbf{J}}_{k(\cdot)}}, \mathbf{U}_{\hat{\mathbf{I}}_{k(\cdot)}}\mathbf{\Sigma}_{\hat{\mathbf{I}}_{k(\cdot)}}$, respectively. Data-specific joint and individual variable loadings are $\mathbf{V}_{\hat{\mathbf{J}}_{k(\cdot)}}, \mathbf{V}_{\hat{\mathbf{I}}_{k(\cdot)}}$, respectively.

For AJIVE estimates, define $\mathbf{C}_{(A)} = [\mathbf{U}_1, \mathbf{U}_2]$ as in section A.1.1. For R.JIVE, let $\mathbf{C}_{(R)} = [\hat{\mathbf{J}}_{1(R)}, \hat{\mathbf{J}}_{2(R)}]$. Compute the rank r_J SVD of $\mathbf{C}_{(\cdot)}$:

$$\mathbf{C}_{(\cdot)} = \mathbf{U}_{\mathbf{J}_{(\cdot)}}\mathbf{\Sigma}_{\mathbf{J}_{(\cdot)}}\mathbf{V}_{\mathbf{J}_{(\cdot)}}^\top.$$

The common normalized joint scores are defined as $\mathbf{U}_{\mathbf{J}_{(\cdot)}}$. The common normalized variable loadings for each dataset are given by $\mathbf{J}_{k(\cdot)}^\top\mathbf{U}_{\mathbf{J}_{(\cdot)}}$.

Web Appendix A.3: Proof of Equivalence between AJIVE and CCA Estimators

THEOREM S0.2. *Let the columns of* $\mathbf{U}_1$ *and* $\mathbf{U}_2$ *represent orthonormal bases for the signal subspaces of* $\mathbb{R}^n$ *for* $\mathbf{X}_1$ *and* $\mathbf{X}_2$*, respectively. The* i^{th} *joint subject score from AJIVE analysis,* $\boldsymbol{u}_{Ji}$*, is given by the* i^{th} *column of* $\mathbf{U}_\mathbf{J}$*, the left singular vectors of* $\mathbf{C} = [\mathbf{U}_1, \mathbf{U}_2]$ *with singular value* σ_{Ji}*. Let* $\omega_{1,i}$ *and* $\omega_{2,i}$*, represent the canonical loadings of the signal subspaces, respectively. Then*

$$\boldsymbol{u}_{Ji} = \frac{1}{\sqrt{2}\sigma_{Ji}}(\mathbf{U}_1\omega_{1,i} + \mathbf{U}_2\omega_{2,i}).$$

That is, AJIVE estimates of joint subject scores are equivalent to a scaled average of the jth *canonical variates of the signal subspaces.*

PROOF. Let $\hat{\mathbf{G}}_k = \mathbf{U}_k \mathbf{\Sigma}_k \mathbf{V}_k^\top$, where $\hat{\mathbf{G}}_k$ is the estimated signal matrix for the kth dataset with rank = $r_k < min(n, p_k)$. Note that $\mathbf{U}_k$ represents the scores from the PCA of the kth dataset. We first consider the first canonical variables. Define the first canonical loadings of the PC scores:

$$\{\hat{\omega}_{1,1},\ \hat{\omega}_{2,1}\} = \underset{\omega_1 \in \mathbb{R}^{r_1}, \omega_2 \in \mathbb{R}^{r_2}, ||\omega_1|| = ||\omega_2|| = 1}{\text{argmax}} \omega_1^\top \mathbf{U}_1^\top \mathbf{U}_2 \omega_2.$$

Since $\mathbf{U}_1$ and $\mathbf{U}_2$ are both column centered and orthonormal, their variance-covariance matrices are given by $\mathbf{\Sigma}_{11} = \mathbf{U}_1^\top \mathbf{U}_1 = \mathbf{I}_{r_1}$ and $\mathbf{\Sigma}_{22} = \mathbf{U}_2^\top \mathbf{U}_2 = \mathbf{I}_{r_2}$, respectively, where $\mathbf{I}_m$ represents the $m \times m$ identity matrix. Similarly, their covariance is given by $\mathbf{\Sigma}_{12} = \mathbf{U}_1^\top \mathbf{U}_2$. Mardia et al. (1979) provides a closed form solution for the loadings $\hat{\omega}_{1,1},\ \hat{\omega}_{2,1}$ as the first left and first right singular vectors, respectively, of $\mathbf{\Sigma}_{11}^{-1/2} \mathbf{\Sigma}_{12} \mathbf{\Sigma}_{22}^{-1/2} = \mathbf{U}_1^\top \mathbf{U}_2$, up to sign. That is, $\hat{\omega}_{1,1}$ is equivalent to the first left singular vector of $\mathbf{U}_1^\top \mathbf{U}_2$, and $\hat{\omega}_{2,1}$ is equivalent to the first right singular vector, where their signs are chosen so that the singular values of $\mathbf{U}_1^\top \mathbf{U}_2$ are all positive. Then the canonical variables are $\hat{\boldsymbol{c}}_{1,1} = \mathbf{U}_1 \hat{\omega}_{1,1}$ and $\hat{\boldsymbol{c}}_{2,1} = \mathbf{U}_2 \hat{\omega}_{2,1}$, and note $||\hat{\boldsymbol{c}}_{1,1}|| = 1$. Define the first canonical correlation $\rho_1 = \hat{\boldsymbol{c}}_{1,1}^\top \hat{\boldsymbol{c}}_{2,1}$. Consider the matrix of concatenated PC scores $[\mathbf{U}_1, \mathbf{U}_2] \in \mathbb{R}^{n \times r_1 + r_2}$.

Then define

$$\hat{\boldsymbol{a}}_1 = [\mathbf{U}_1, \mathbf{U}_2][\hat{\omega}_{1,1}^\top, \hat{\omega}_{2,1}^\top]^\top = \hat{\boldsymbol{c}}_{1,1} + \hat{\boldsymbol{c}}_{2,1}.$$

Next we show that $\hat{\boldsymbol{a}}_1$ is equivalent to the JIVE solution by showing $\hat{\boldsymbol{a}}_1$ is the first left singular vector of $\mathbf{C} = [\mathbf{U}_1, \mathbf{U}_2]$ up to scaling. Let $\omega^* = [\hat{\omega}_{1,1}^\top, \hat{\omega}_{2,1}^\top]^\top$. Since $\hat{\omega}_{1,1}$ and $\hat{\omega}_{2,1}$ are left and right singular vectors of $\mathbf{U}_1^\top \mathbf{U}_2$, respectively, we have $\mathbf{U}_1^\top \mathbf{U}_2 \hat{\omega}_{2,1} = \rho_1 \hat{\omega}_{1,1}$ and $\mathbf{U}_2^\top \mathbf{U}_1 \hat{\omega}_{1,1} = \rho_1 \hat{\omega}_{2,1}$. Additionally, $\mathbf{C}^\top \mathbf{C} = \begin{bmatrix} \mathbf{I} & \mathbf{U}_1^\top \mathbf{U}_2 \\ \mathbf{U}_2^\top \mathbf{U}_1 & \mathbf{I} \end{bmatrix} = \mathbf{I} + \begin{bmatrix} \mathbf{0} & \mathbf{U}_1^\top \mathbf{U}_2 \\ \mathbf{U}_2^\top \mathbf{U}_1 & \mathbf{0} \end{bmatrix}$. Thus,

$$\begin{aligned} \mathbf{C}^\top \mathbf{C} \omega^* &= \omega^* + \begin{bmatrix} \mathbf{U}_1^\top \mathbf{U}_2 \hat{\omega}_{2,1} \\ \mathbf{U}_2^\top \mathbf{U}_1 \hat{\omega}_{1,1} \end{bmatrix} \\ &= (1 + \rho_1)\omega^*. \end{aligned}$$

Hence, $\omega^*/||\omega^*|| = \omega^*/\sqrt{2}$ is the first normalized eigenvector of $\mathbf{C}^T\mathbf{C}$, which is the first right singular vector of $\mathbf{C}$. Let $\mathbf{C} = \mathbf{U_J} \mathbf{\Sigma_J} \mathbf{V_J}^\top$ be the SVD of $\mathbf{C}$, wherein the first r_J columns of $\mathbf{U_J}$ are the joint components from the JIVE decomposition and $\mathbf{\Sigma_J}$ has diagonal elements σ_j. Let $\hat{\boldsymbol{u}}_{J,1}$ be the first joint component. Note the first row of $\mathbf{V_J}^\top$ is equal to ω^*. Then

$$\begin{aligned} \mathbf{C}\frac{\omega^*}{\sqrt{2}} &= \frac{1}{\sqrt{2}}[\mathbf{U}_1, \mathbf{U}_2][\hat{\omega}_{11}^\top, \hat{\omega}_{21}^\top]^\top \\ &= \frac{\hat{\boldsymbol{c}}_{1,1} + \hat{\boldsymbol{c}}_{2,1}}{\sqrt{2}} \\ &= \sigma_1 \hat{\boldsymbol{u}}_{J,1} \\ &= \sqrt{(1 + \rho_1)}\hat{\boldsymbol{u}}_{J,1} \end{aligned}$$

which corresponds to the first joint component form the AJIVE decomposition and therefore leads to the following equivalence between common scores and canonical variables:

$$\hat{\boldsymbol{u}}_{J,1} = \frac{\hat{\boldsymbol{c}}_{1,1} + \hat{\boldsymbol{c}}_{2,1}}{\sqrt{2(1+\rho_1)}}.$$

A similar argument applies to the other joint components.

WEB APPENDIX B: SIMULATION STUDY

A simulation study was conducted according to a 2^3 full factorial design, in order to examine the effectiveness of AJIVE and R.JIVE for estimating the JIVE model and our proposed method for estimating the number of joint components the JIVE model.

In order to achieve the pre-described proportions of variance explained via joint and individual signals, we derive numerical solutions to:

$$R^2_{Ik} = \frac{c_k^2 \mathrm{tr}(\mathbf{A}_k \mathbf{A}_k^\top)}{c_k^2 \mathrm{tr}(\mathbf{A}_k \mathbf{A}_k^\top) + 2c_k \mathrm{tr}(\mathbf{A}_k \mathbf{E}^\top) + d_k^2 \mathrm{tr}(\mathbf{J}_k \mathbf{J}_k^\top) + \mathrm{tr}(\mathbf{E}_k \mathbf{E}_k^\top) + 2d_k \mathrm{tr}(\mathbf{J}_k \mathbf{E}_k^\top)}$$

$$R^2_{Jk} = \frac{d_k^2 \mathrm{tr}(\mathbf{J}_k \mathbf{J}_k^\top)}{d_k^2 \mathrm{tr}(\mathbf{J}_k \mathbf{J}_k^\top) + 2d_k \mathrm{tr}(\mathbf{J}_k \mathbf{E}^\top) + c_k^2 \mathrm{tr}(\mathbf{A}_k \mathbf{A}_k^\top) + \mathrm{tr}(\mathbf{E}_k \mathbf{E}_k^\top) + 2c_k \mathrm{tr}(\mathbf{A}_k \mathbf{E}_k^\top)}$$

Average computation times (Web Table S1) show that CJIVE Computes solutions between twice and 100 times as faster than AJIVE or R.JIVE.

Accuracy of joint rank selection is discussed in the main manuscript. However, R.JIVE also chooses individual signal ranks, and therefore total ranks. Web Figure S1 exhibits the total ranks chosen via R.JIVE. Notably, total ranks are nearly always underestimated when joint variation is small in both datasets ($R^2_{J1} = R^2_{J2} = 0.05$). On the other hand, the total ranks are nearly always correctly selected when $R^2_{J1} = R^2_{J2} = 0.5$. When $p_2 = 10,000$ (panel B), total rank selection is also accurate for cases with $R^2_{J1} == 0.5$ and $R^2_{J2} = 0.05$.

Table S1. Mean (S.D.) computation run-times from simulation study (in minutes)

R^2_{J1}	R^2_{J2}	p_2	CJIVE-r_k	CJIVE-Over	AJIVE-r_k	AJIVE-Over	R.JIVE-Free	R.JIVE-Oracle
0.05	0.05	200	<0.1 (<.1)	<0.1 (<.1)	<0.1 (<.1)	<0.1 (<.1)	0.5 (0.35)	0.1 (0.06)
0.5	0.05	200	<0.1 (<.1)	<0.1 (<.1)	<0.1 (<.1)	<0.1 (<.1)	0.4 (0.21)	2.1 (1.72)
0.05	0.5	200	<0.1 (<.1)	<0.1 (<.1)	<0.1 (<.1)	<0.1 (<.1)	0.4 (0.11)	1.7 (1.36)
0.5	0.5	200	<0.1 (<.1)	<.1 (<.1)	<0.1 (<.1)	<0.1 (<.1)	0.1 (0.01)	0.5 (0.67)
0.05	0.05	10000	0.1 (0.01)	<0.1 (0.01)	0.3 (0.05)	0.1 (0.03)	0.3 (0.1)	1.6 (0.41)
0.5	0.05	10000	0.1 (0.01)	<0.1 (0.01)	0.3 (0.03)	0.2 (0.02)	0.4 (0.15)	3.8 (0.84)
0.05	0.5	10000	<0.1 (0.02)	<0.1 (0.02)	0.2 (0.08)	0.1 (0.05)	0.3 (0.13)	2.6 (2.09)
0.5	0.5	10000	0.1 (0.01)	<0.1 (0.01)	0.3 (0.03)	0.2 (0.02)	0.1 (0.01)	2.8 (0.53)

Both AJIVE and CJIVE require estimation of total ranks prior to analyses. To examine how well scree plot estimation works in our simulation settings, we recorded eigenvalues of simulated data when $R^2_{J1} = R^2_{J2} = 0.05$. This is the most challenging setting since they have the least amount of variance, and thus the elbow of the scree plot is least distinct among the simulation scenarios. For $p_2 = 200$, overall the fifth eigenvalue (i.e., smallest signal eigenvalue) is distinguishable from the noise eigenvalues, as the noise values follow linear rather than exponential decay (Figure S2, panel A). For $p_2 = 10000$, the fifth eigenvalue is clearly distinguishable, such that we can assume AJIVE-r_k is equivalent to AJIVE-Scree plot, and similarly, CJIVE-r_k is equivalent to CJIVE-Scree plot (Figure S2, panel B).

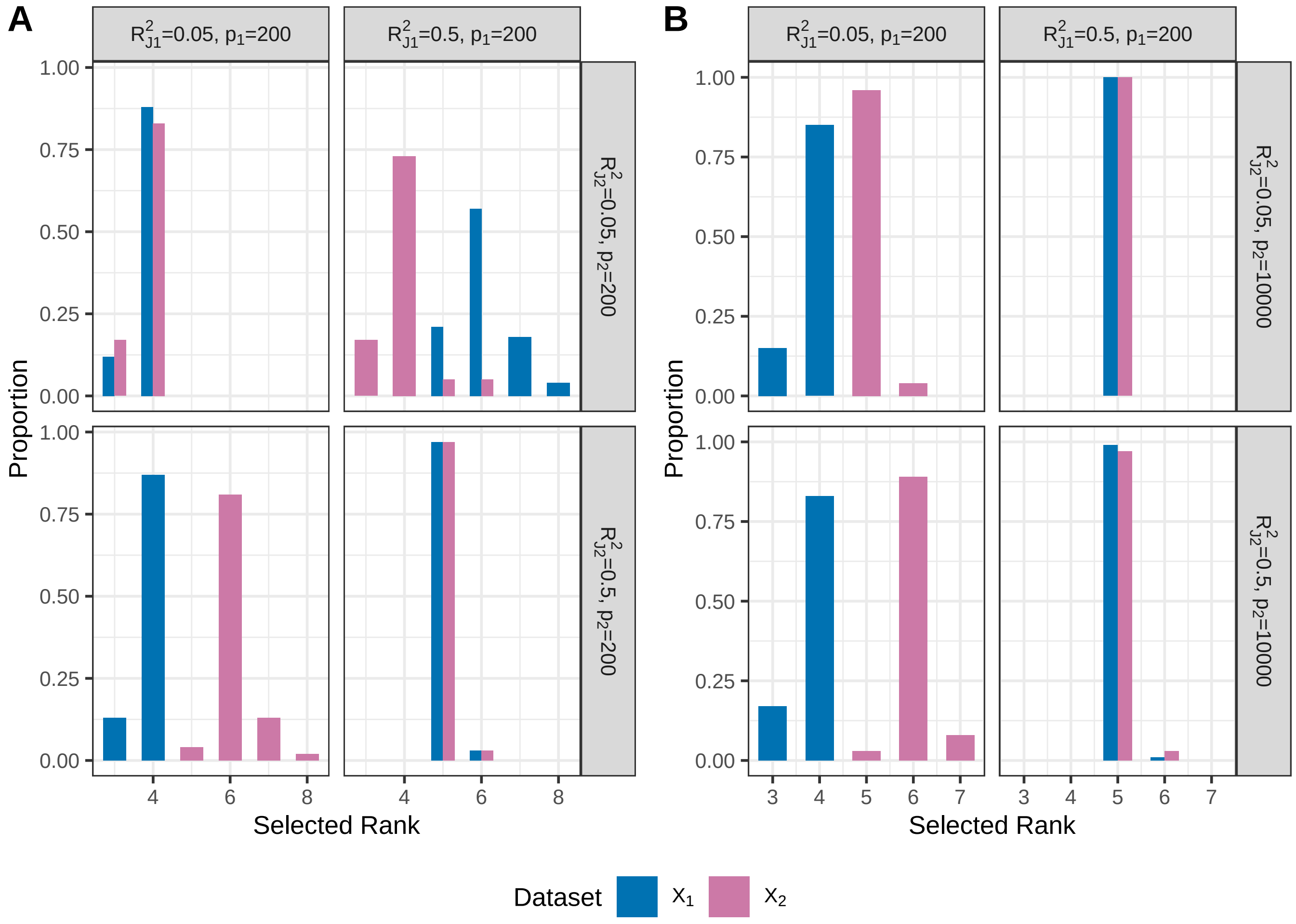

Figure S1. Total rank (signal rank) estimates from R.JIVE. True ranks are $r_1 = r_2 = 5$ in both (A) and (B).

WEB APPENDIX C: HUMAN CONNECTOME PROJECT

The main article uses JIVE to simultaneously examine functional connectivity arising from resting-state fMRI scans and structural connectivity from diffusion-weighted MRI scans in the Human Connectome Project. Specifically, we used resting-state scans of the name rfMRI_REST1_LR_Atlas_hp2000_clean.dtseries.nii with subject-specific Desikan labels from

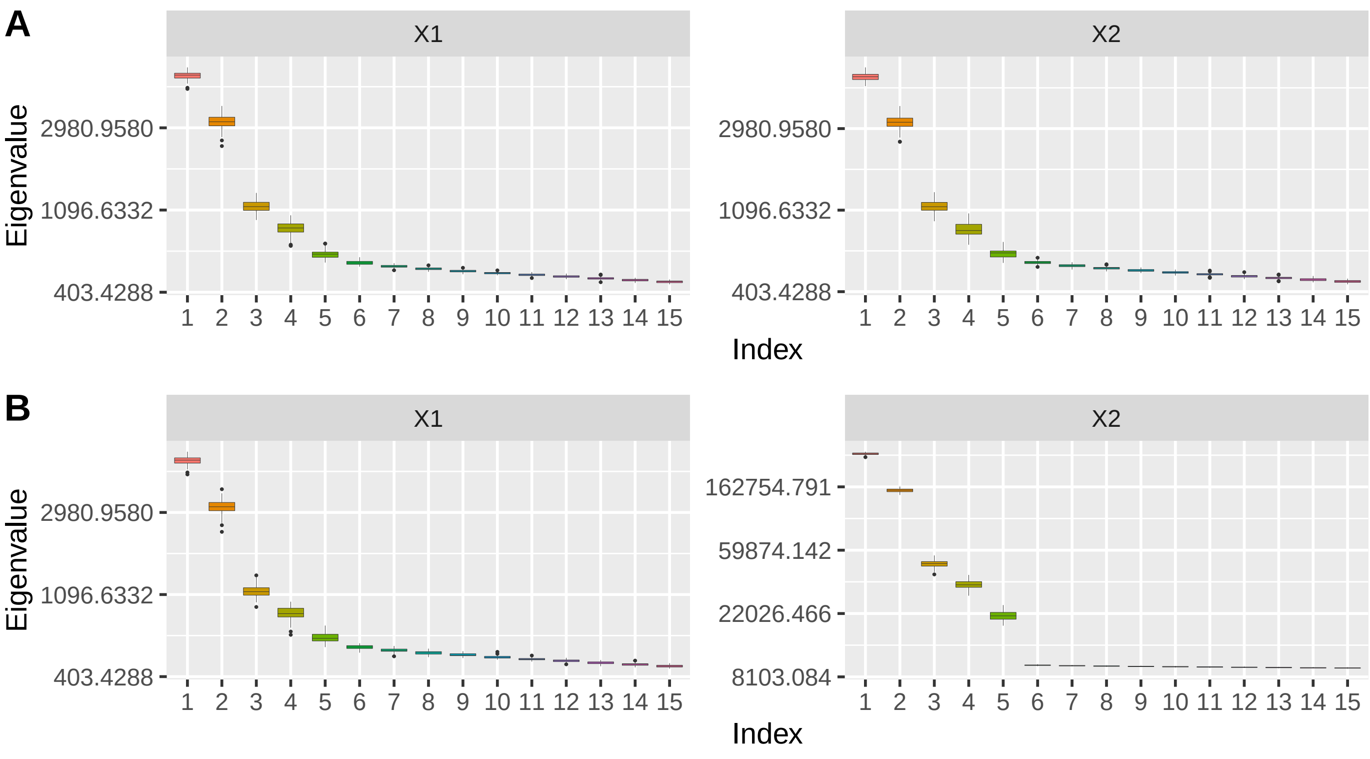

Figure S2. Boxplots of the first 15 singular values from pairs of simulated datasets, where $R^2_{J1} = R^2_{J2} = 0.05$ with $p_2 = 200$ (A) and $R^2_{J1} = R^2_{J2} = 0.05$ with $p_2 = 10,000$ (B).

<subj>.aparc.32k_fs_LR.dlabel.nii. Additional details are in the main manuscript. Here we present the images of mean SC and FC networks (Web Figure S3) and a summary of demographics (Web Table 2).

Web Appendix C.1: Dimension Selection

Table S3 displays the percent variance by PCA at different ranks for each dataset.

Total signal ranks (the total number of joint and individual components) were chosen using four methods: 1) "elbow" method of scree plot, 2) the respective number of eigenvalues which account for 80% and 95% of the sum of eigenvalues, 3) R.JIVE permutation tests, and 4) via an irregular grid search. It is notable that the joint rank chosen by AJIVE and CJIVE depends on the total rank chosen initially.

Web Appendix C.2: CJIVE Variable Loadings

Web Figure 3 displays heatmaps and L1 norms of loadings onto the second component of the joint subspace for each data block. The second joint component was not statistically associated with fluid

Table S2. Demographics of HCP Imaging Data

**Descriptive Statistics (n = 998)**					
Age		***Sex***		***Fluid Intelligence (gF)***	
21-25:	218 (21.8%)	*Female*:	532 (53.3%)	*Mean*:	17.04
26-30:	429 (43.0%)	*Male*:	466 (46.7%)	*S.D*:	4.70
31+:	351 (35.2%)			*Median*:	18.0

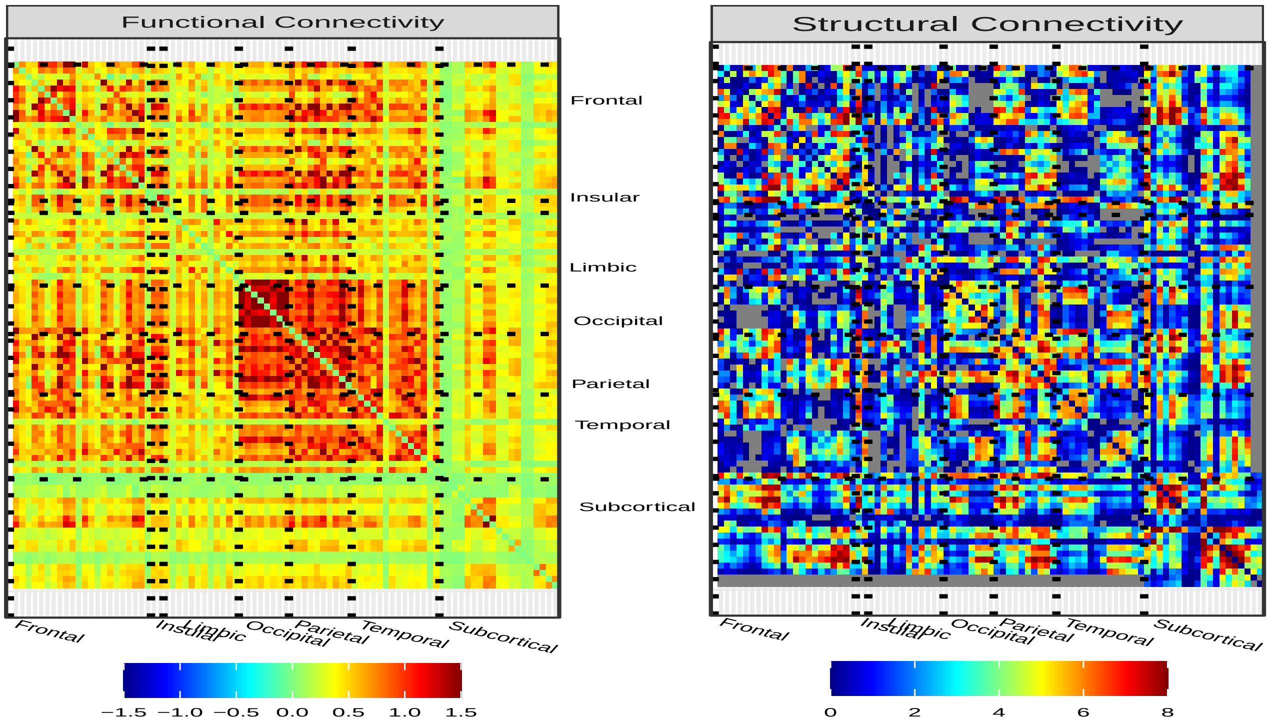

Figure S3. Mean functional connectivity (Fisher z-transformed correlations, left) and structural connectivity (log streamline counts, right) for the n = 998 HCP participants with data from both DTI and rs-fMRI available.

intelligence (gF). However, the hemispheric symmetry of the 75th percentile of the loadings' L1 norms reveal biological relevance.

Web Figure S5 displays individual loadings for FC. The first component's largest values occur for edges connecting cortical regions, while the largest values for components 2 and 3 occur for edges connecting cortical and subcortical ROIs.

Individual loadings for SC, displayed in Web Figure S6, are much more sparse than loadings onto the first component of the joint subspace.

REFERENCES

Feng, Q., Jiang, M., Hannig, J., and Marron, J. S. (2018). Angle-based joint and individual variation explained. *Journal of Multivariate Analysis*, 166:241–265.

Lock, E. F., Hoadley, K. A., Marron, J. S., and Nobel, A. B. (2013). Integrated analysis of multiple data types. *Annals of Applied Statistics*, 7(1):523–542.

Mardia, K.V. (University of Leeds, U., Kent, J. T. (University of Leeds, U., and Bibby, J. M. T. O. U. (1979). *Multivariate analysis*. Acadmic Press, Inc., 10 edition.

Wedin, P. A. (1972). Perturbation bounds in connection with singular value decomposition. *BIT Numerical Mathematics*, 12(1):99–111.

Table S3. : Chosen ranks and corresponding proportions of variation explained in the FC (Pearson correlations) and SC (streamline counts) derived from HCP data. Entries of "–" indicate that the given rank was not used for the given modality. Abbreviations: FC = Functional Connectivity SC = Structural Connectivity

Signal Rank	FC (%) Variance Explained	SC (%) Variance Explained
2	53.84	9.12
5	59.69	16.61
7	62.48	19.51
10	65.62	23.08
15	69.75	27.36
20	72.89	30.82
25	75.4	33.62
30	77.42	36.05
39	80.25	–
40	80.53	40.17
50	82.85	43.6
75	86.79	50.31
100	89.32	55.43
200	94.32	69.11
225	95.01	–
330	–	80.03
500	98.57	89.08
683	–	95.09
990	99.99	99.95

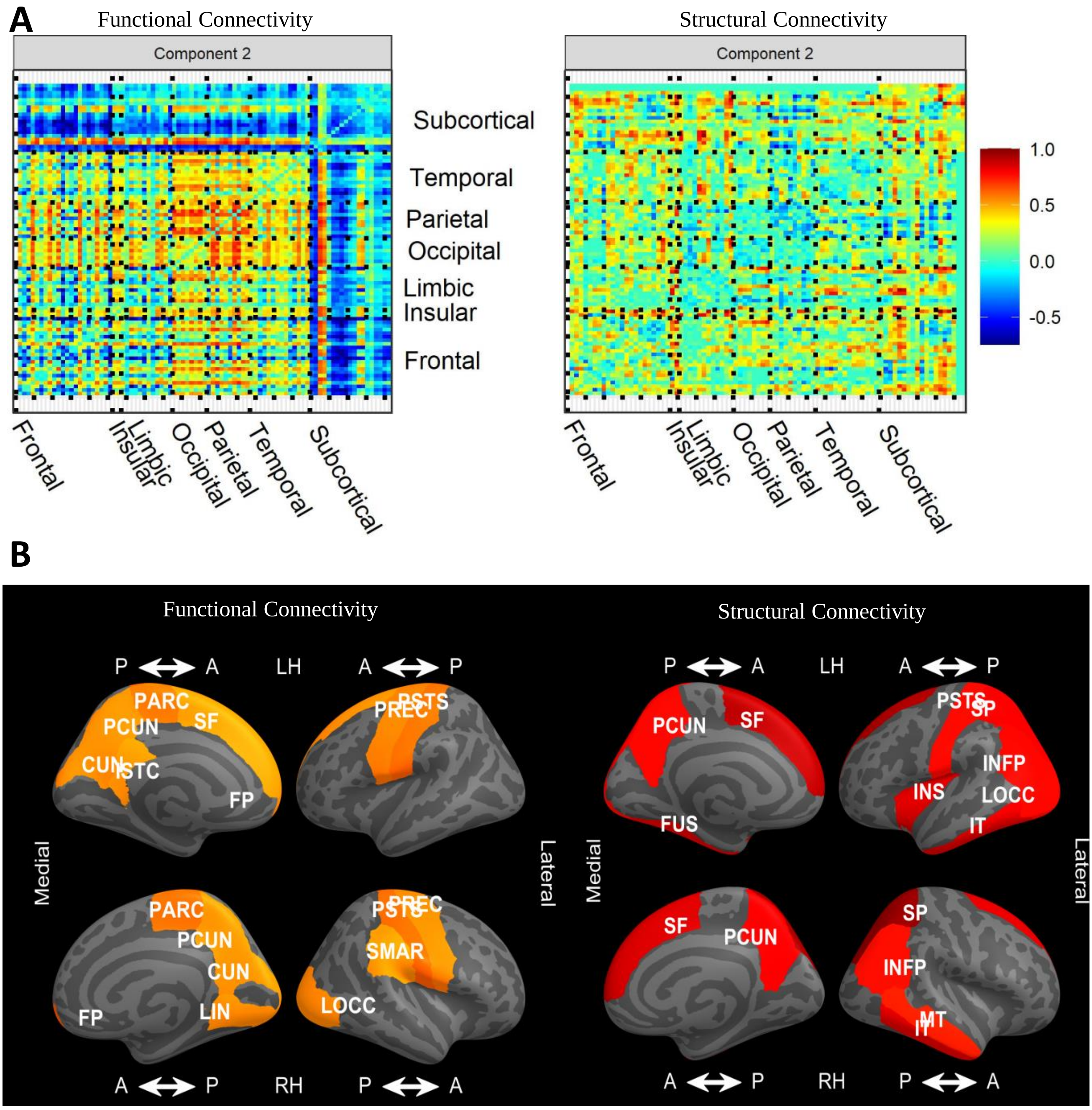

Figure S4. (A) Variable loadings for the second component of the joint signal space estimated by CJIVE and displayed on heatmaps. Sub-figure (B) displays the top 25th percent of L1 norms of the variable loadings related to each cortical ROI for joint component 1. L1 norm for an ROI equals the sum of the absolute values of the rows of (a), excluding subcortical regions. Abbreviations: BSTS: Bank of STS, CAC: Caudal anterior cingulate, CMF: Caudal middle frontal, FP: Frontal pole, ISTC: Isthmus cingulate, LOCC: Lateral occipital, MORB: Medial orbito-frontal, MT: Middle temporal, PARH: Parahippocampal, PCAL: Pericalcarine, PCUN: Precuneus, PREC: Precentral, PSTS: Postcentral, SMAR: Supramarginal, ST: Superior temporal

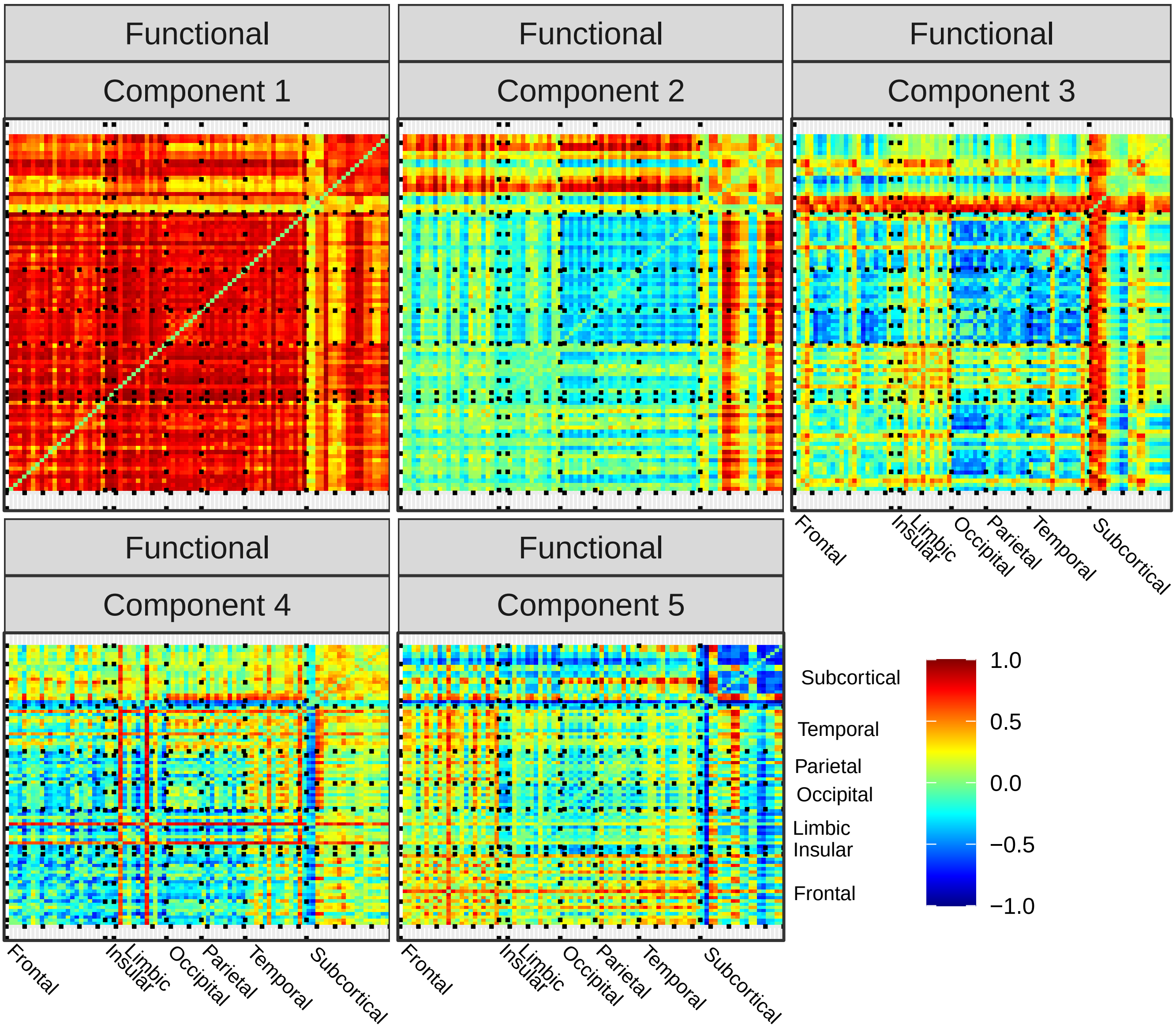

Figure S5. Heatmaps of variable loadings for each component of the FC individual subspace.

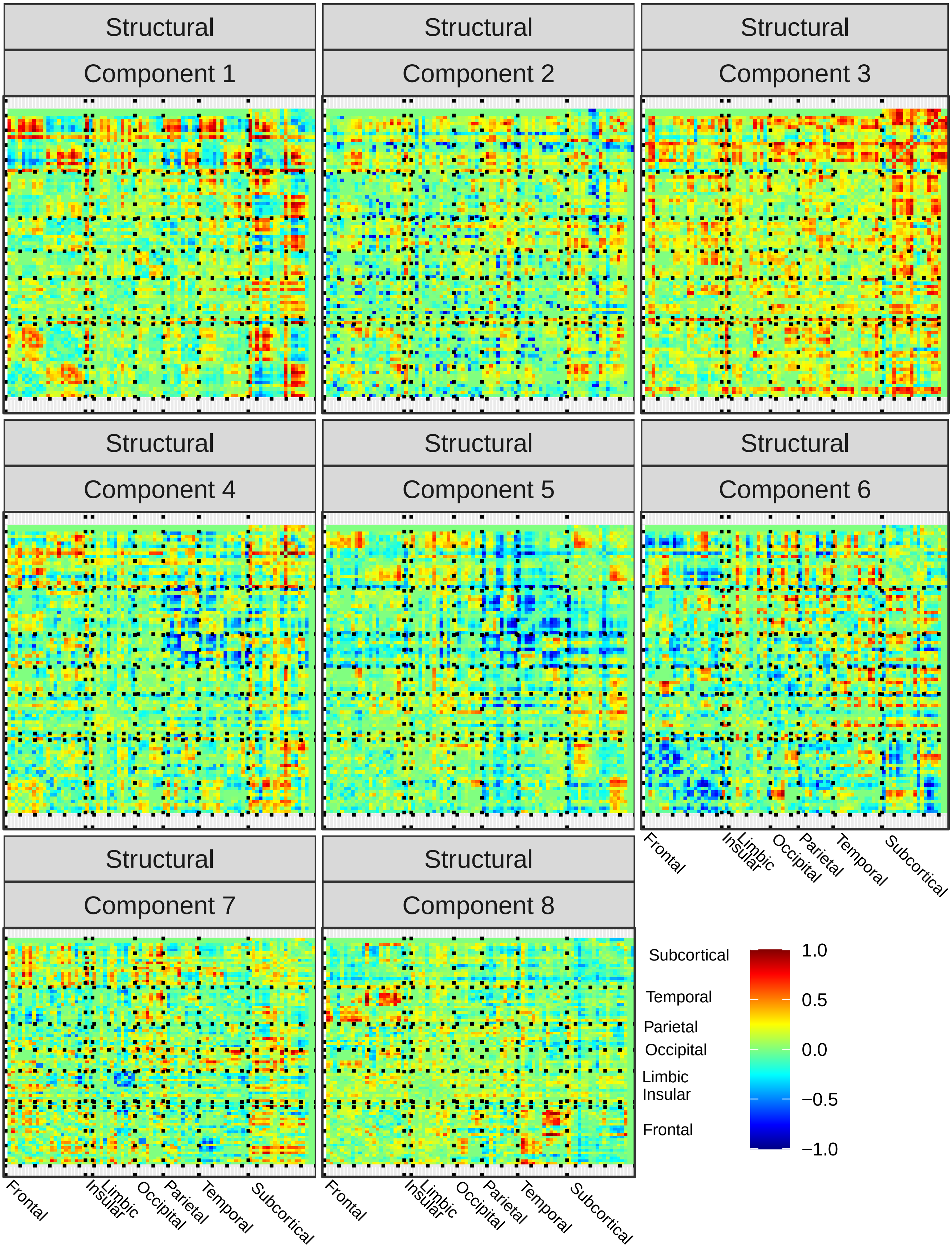

Figure S6. Heatmaps of variable loadings for each component of the SC individual subspace.
